# Assessment of respiratory and systemic toxicity of Benzalkonium chloride following a 14-day inhalation study in rats

**DOI:** 10.1186/s12989-020-0339-8

**Published:** 2020-01-28

**Authors:** Hye-Yeon Choi, Yong-Hoon Lee, Cheol-Hong Lim, Yong-Soon Kim, In-Seop Lee, Ji-Min Jo, Ha-Young Lee, Hyo-Geun Cha, Hee Jong Woo, Dong-Seok Seo

**Affiliations:** 1 Inhalation Toxicity Research Center, Occupational Safety and Health Research Institute, KOSHA, 30 Expo-ro 339beon-gil, Yuseong-gu, Daejeon, 34122 Republic of Korea; 2 Laboratory of Immunology, College of Veterinary Medicine, Seoul National University, 1 Gwanak-ro, Gwanak-gu, Seoul, 08826 Republic of Korea

**Keywords:** Benchmark dose, BAC(BKC), Derived no-effect level, Inhalation, Toxicity

## Abstract

**Background:**

Although biocides at low concentrations have been used to control pests, they can be more harmful than industrial chemicals as humans are directly and frequently exposed to such biocides. Benzalkonium chloride (BAC or BKC) is a non-toxic substance used to control pests. Recently, BAC has been increasingly used as a component in humidifier disinfectants in Korea, raising a serious health concern. Moreover, it poses significant health hazards to workers handling the chemical because of direct exposure. In the present study, we aimed to evaluate the respiratory toxicity of BAC due to its inhalation at exposure concentrations of 0.8 (T1 group), 4 (T2 group) and 20 (T3 group) mg/m^3^.

**Results:**

In our previous study on the acute inhalational toxicity of BAC, bleeding from the nasal cavity was observed in all the rats after exposure to 50 mg/m^3^ BAC. Therefore, in this study, 20 mg/m^3^ was set as the highest exposure concentration, followed by 4 and 0.8 mg/m^3^ as the medium and low concentrations for 6 h/day and 14 days, respectively. After exposure, recovery periods of 2 and 4 weeks were provided. Additionally, alveolar lavage fluid was analyzed in males of the BAC-exposed groups at the end of exposure and 2 weeks after exposure to evaluate oxidative damage.

In the T3 group exposed to BAC, deep breathing, hoarseness, and nasal discharge were observed along with a decline in feed intake and body weight, and nasal discharge was also observed in the T1 and T2 groups. ROS/RNS, IL-1β, IL-6, and MIP-2 levels decreased in a concentration-dependent manner in the bronchoalveolar lavage fluid. Histopathological examination showed cellular changes in the nasal cavity and the lungs of the TI, T2, and T3 groups.

**Conclusions:**

As a result, it was confirmed that the target organs in the respiratory system were the nasal cavity and the lungs. The adverse effects were evaluated as reversible responses to oxidative damage. Furthermore, the no observed adverse effect level was found to be less than 0.8 mg/m^3^ and the lowest benchmark dose was 0.0031 mg/m^3^. Accordingly, the derived no-effect level of BAC was calculated as 0.000062 mg/m^3^.

## Introduction

Biocides are non-agricultural pesticides used to control, eliminate, inhibit, detoxify, or prevent harmful organisms in ways other than mere physical and mechanical actions. They include active materials such as microorganisms and substances that affect harmful organisms. A product containing one or more of these active substances is called a biocidal product.

Currently, biocides are used extensively. In 2011, the biocides caused several social problems in Korea because of unexplained lung disease in pregnant women and infants due to exposure to humidifier disinfectants. Furthermore, these biocides are predicted to cause more severe lung diseases among workers handling such substances than the general public due to direct and longer exposure to biocides at a high concentration in the work environment. However, currently, there is a lack of research in this regard.

Benzalkonium chloride (BAC) was classed as a Category III antiseptic active ingredient by the US FDA because of a lack of adequate safety data for its use as both a health care antiseptic and consumer antiseptic product [1]. BAC is a mixture of alkylbenzyl dimethylammonium chlorides of various even-numbered alkyl chain lengths (C8-C18) [2] and it is commonly used as disinfectants in food, industrial and domestic areas [3]. The greatest biocide activity is associated with the C12-C14 derivatives, which are the main components of the mixture [2]. In general, the n-C12 homolog is most effective against yeast and fungi, and the n-C14 homolog against gram-positive bacteria [4]. The mode of action of quaternary ammonium compounds appears to be associated with their effect on the cytoplasmic membrane, which controls cell permeability [3]. Furthermore, BAC has also been used as an active ingredient (amount contained in product: 0.045%, *w/v*) in humidifier disinfectants. It is mainly used by diluting with water in an ultrasonic-type humidifier, and therefore, it can be inhaled in the form of aerosol generated into the atmosphere. Therefore, BAC is suspected as a cause of the social disaster related to humidifier disinfectant in Korea.

BAC is an active ingredient in several consumer products, including pharmaceutical products such as eye, ear, and nasal drops or sprays, as a preservative; personal care products such as hand sanitizers, wet wipes, shampoos, deodorants, and cosmetics; skin antiseptics, such as Bactine and Dettol; throat lozenges [5] and mouthwashes, as a biocide; spermicidal creams; over-the-counter single-application treatments for herpes, cold-sores, and fever blisters, such as RELEEV and Viroxyn; burn and ulcer treatments; spray disinfectants for hard surface sanitization; cleaners for floor and hard surfaces as a disinfectant, such as Lysol; algaecides for clearing algae, moss, and lichens from paths, roof tiles, swimming pools, and masonry. BAC is also used in several non-consumer processes and products; for example, as an active ingredient in surgical disinfection. A comprehensive list of uses includes industrial applications [6]. Benzalkonium chloride is a frequently used preservative in eye drops; typical concentrations range from 0.004 to 0.01%. Higher concentrations can be caustic [7] and can cause irreversible damage to the corneal endothelium [8]. Occupational exposure to BAC has been linked to the development of asthma [9]. Furthermore, BAC has been commonly used as a pharmaceutical preservative and antimicrobial since the 1940s. Therefore, there is a need for studies to evaluate the effects of occupational exposure to BAC in the form of aerosols, considering the associated health risks.

Benzalkonium chloride (CAS Registry Number 8001-54-5) was considered for this study because it complies with the following five criteria for selecting substances for occupational exposure assessment of biocides [10].

1. The products containing BAC have a high frequency of use and BAC is identified as hazardous by the Korea Institute of Disease Control.
2. The substance is included in the designated substances of EE Directive 98/8/EC Annexes 1 and 1A, and is distributed in Korea.
3. The substance has been used frequently in traditional workplaces and whose health risks have been reported, but has poor or no exposure assessment data.
4. Data on the distribution quantity or import volume is officially announced in the domestic distribution quantity statistics data.
5. Substance that is included in the above four items and is capable of causing health hazards due to occupational exposure in the form of an aerosol.

Although several previous studies on the toxicity of BAC have been conducted individually, there is a lack of toxicity studies on systemic inhalation exposure in accordance with the OECD GLP (*Good Laboratory Practice*) Test Guidelines. Therefore, in this study, we evaluated the toxicity of respiratory exposure to BAC in rats using a whole body chamber system and mist-generating system. The rats were repeatedly exposed to aerosols for 2 weeks to assess the toxic response induced by BAC, and the recovery period of 2 and 4 weeks was included to evaluate the reversibility of the induced toxic response and to compare and analyze related cytokines.

## Results

### Concentration and particle size distribution of BAC in the whole-body exposure chamber

**Fig. 1:**
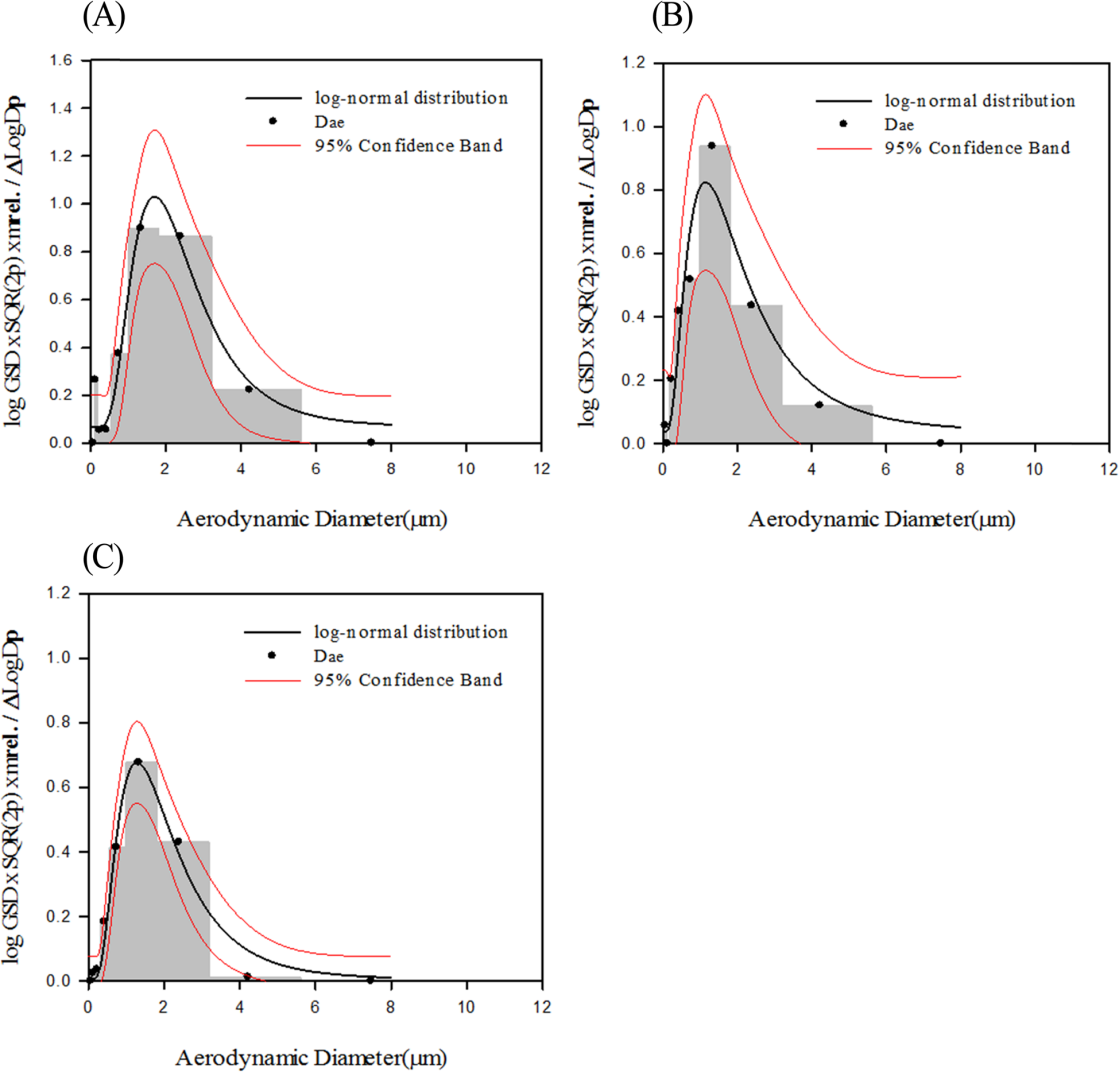
Particle size distributions of BAC in the chambers. 0.8 mg/m^3^(**a**), 4.0 mg/m^3^(**b**), 20 mg/m^3^ (**c**)

The mean concentration of BAC in the whole-body exposure chambers of the T1(0.8 mg/m^3^), T2(4 mg/m^3^) and T3(20 mg/m^3^) groups during the exposure period was 0.84 ± 0.09, 4.01 ± 0.12, and 19.57 ± 0.97 mg/m^3^, respectively, the MMAD of the aerosols was 1.614, 1.090, and 1.215 μm, respectively, and the GSD was 2.00, 1.86, and 1.51, respectively. The MMAD and GSD were confirmed to be within the range recommended by the Organization for Economic Cooperation and Development (OECD, 2018). The size distribution of BAC aerosol particles is shown in Fig. 1. The T_95_, which is the time to reach 95% concentration of 0.8, 4, and 20 mg/m^3^ BAC in the chamber, was 7.1, 5.7, and 3.4 min, respectively, as measured using a Portable Aerosol Spectrometer (Model 11-A, GRIMM Aerosol Technik GmbH & Co. KG, Germany).

### Clinical signs, body weight, and feed intake

None of the rats in the test groups died during the exposure period. In the T1 group, nasal discharge was observed in two female rats. In the T2 group, nasal discharge was observed in 2 male and 3 female rats. In the T3 group, deep breathing was observed in 1 male rat, rales in 5 male rats, and nasal discharges in 5 female and 5 male rats. Among the general signs observed during the exposure period, soiled perineal region, rales, and discharge were continuously observed during the 2-week recovery period.

**Fig. 2:**
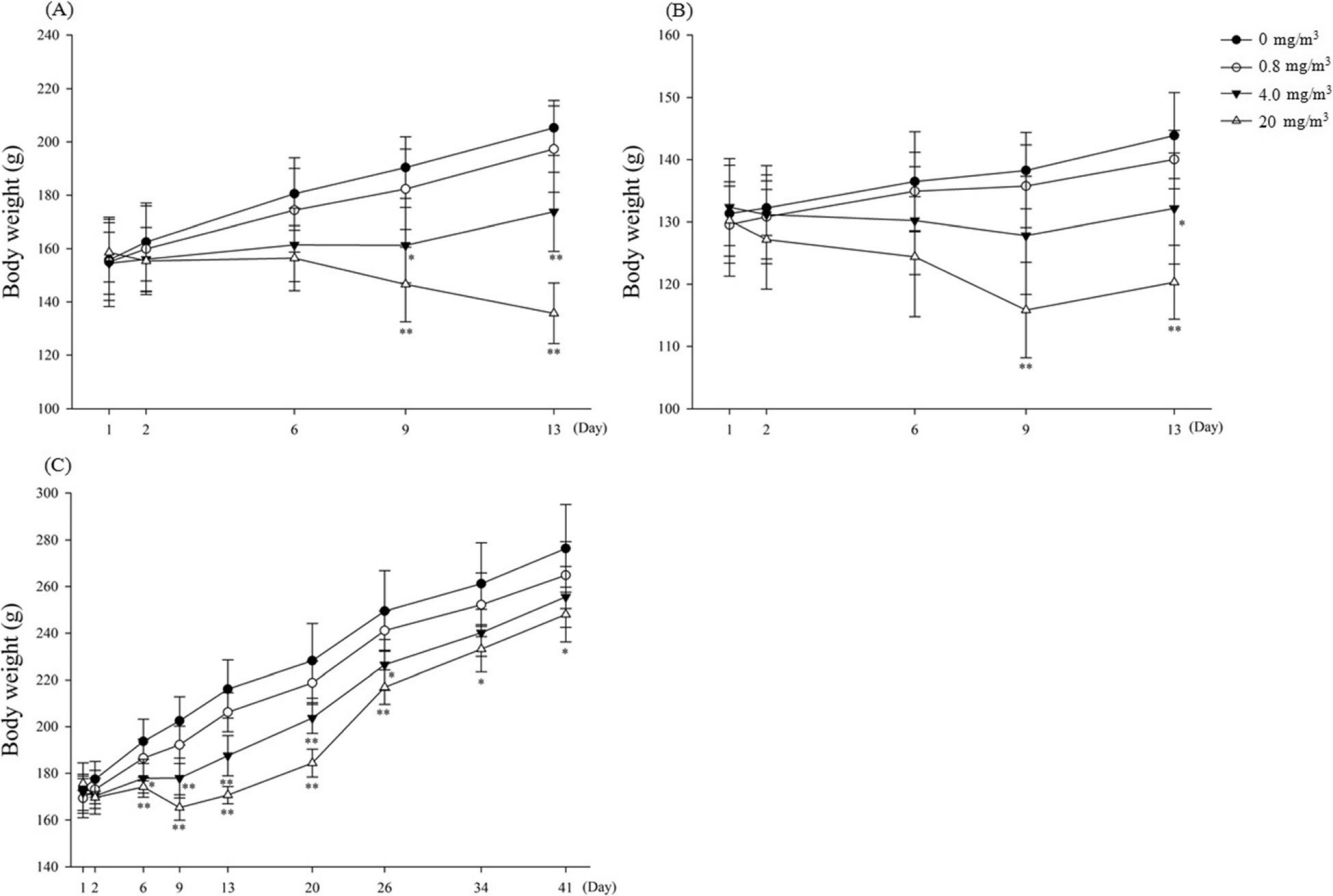
Changes of body weights in the rats exposed to BAC. Males (**a**) and Females (**b**) of the main groups, Males (**c**) of the recovery groups. Significantly different from control by Dunnett test: *p < 0.05, **p < 0.01. Significantly different from control by Dunn Rank Sum test: *p < 0.05, **p < 0.001

With respect to the body weight, the T3 and T2 groups of the male and female rats showed a statistically significant decrease compared with that of the control group rats during the exposure period, but no statistically significant weight loss was observed in the T1 group of male and female rats. In the recovery groups of male rats, statistically significant differences were observed only in the T3 group rats during the recovery period (Fig. 2).

**Fig. 3:**
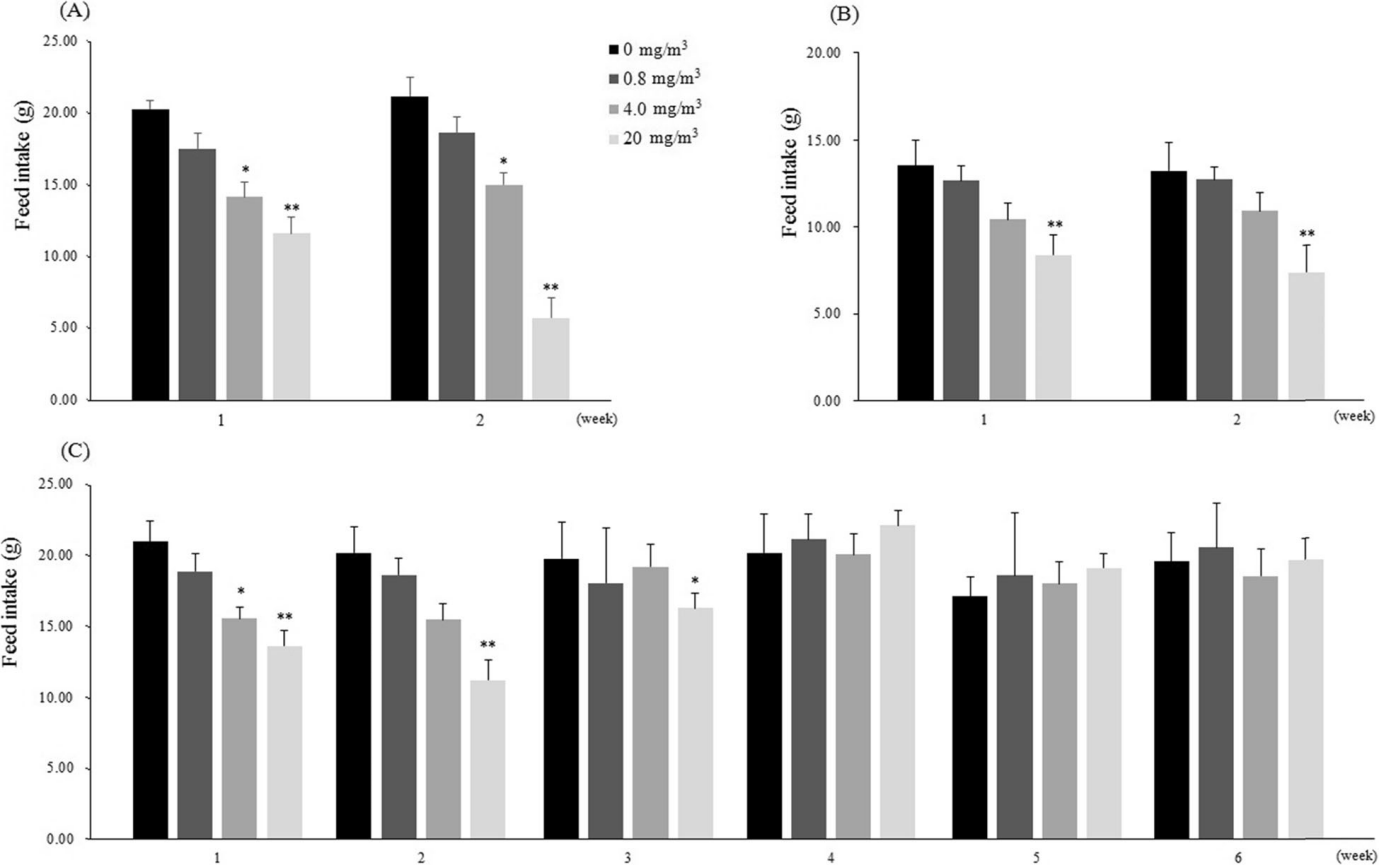
Changes of feed intake in the rats exposed to BAC. Males (**a**) and Females (**b**) in the main groups, Males (**c**) in the recovery groups

During the exposure period, a statistically significant decrease in feed intake was observed in the male rats of the T2 and T3 groups, and in the female rats of the T3 group compared with that in the control group rats. No statistically significant changes in feed intake were observed in the T1 group compared with that in the control group. During the recovery period, a statistically significant reduction in feed intake was observed only at 1 week of recovery in the T3 group, and there was no decrease in feed intake thereafter (Fig. 3).

### Hematology and blood biochemistry

Among the male rats in the main groups, the red blood cell (RBC) count, hematocrit (HCT) level, hemoglobin (Hb), mean corpuscular hemoglobin concentration (MCHC), activated partial thromboplastin time (APTT), and prothrombin time (PT) showed a statistically significant increase compared with those of the control and the mean corpuscular volume (MCV), platelet (PLT) count, absolute count of lymphocyte, absolute and relative counts of reticulocytes showed a statistically significant decrease in the T3 group. The T2 group showed a statistically significant increase in the RBC count, HCT and Hb level, absolute and relative counts of eosinophils and PT, and a statistically significant decrease in the PLT level compared with those of the control group. In the T1 group, a statistically significant increase was observed in the Hb level compared with that in the control. In female rats of the main groups, the results of hematological analysis showed a statistically significant decrease in the WBC count, absolute counts of lymphocyte and monocyte, relative count of reticulocytes compared with those in the control, and a statistically significant increase in the APTT and PT in the T3 group. A statistically significant increase in the PT was observed only in the T2 test group. The remaining parameters showed no statistically significant change (Table 1). After 2 weeks of recovery period, the MCV, absolute and relative counts of reticulocytes, and PLT of the male recovery T3 group showed a statistically significant increase compared with those of the control. The male recovery T2 group showed a statistically significant increase in the absolute and relative counts of reticulocytes and PLT compared with those of the control. The remaining parameters showed no statistically significant change. After 4 weeks of recovery period, the MCV, MCH, and PT of the male recovery T3 group showed a statistically significant increase compared with those of the control (Table 2), but no statistically significant change was observed in the other parameters (Additional file 1: Tables S1 and S2).

**Table 1:** Hematologic and blood chemical parameters of main group rats exposed to benzalkonium chloride

	Main group							
Sex	Male				Female			
Concentration (mg/m^3^)	0	0.8	4	20	0	0.8	4	20
Hematology								
WBC (× 10^3^/μL)	4.17 ± 0.73	4.56 ± 0.46	3.47 ± 0.72	3.07 ± 0.84	4.42 ± 0.59	4.09 ± 0.54	3.55 ± 0.60	2.50 ± 0.39^b^
RBC (×10^6^/μL)	8.40 ± 0.29	8.68 ± 0.16	8.97 ± 0.13^b^	9.56 ± 0.19^b^	9.04 ± 0.21	8.82 ± 0.10	9.21 ± 0.15	9.54 ± 0.37
HCT (%)	43.52 ± 1.27	44.56 ± 1.17	45.42 ± 0.69^a^	48.12 ± 1.16^b^	45.28 ± 0.59	44.48 ± 0.61	46.12 ± 0.76	47.58 ± 2.35
Hb (g/dL)	14.50 ± 0.26	14.96 ± 0.21^a^	15.42 ± 0.30^b^	16.44 ± 0.34^b^	15.60 ± 0.24	15.32 ± 0.42	15.86 ± 0.23	16.40 ± 0.67
MCV (fL)	51.84 ± 1.06	51.30 ± 0.57	50.62 ± 0.59	50.32 ± 0.37^c^	50.14 ± 0.67	50.40 ± 0.46	50.06 ± 0.39	49.88 ± 0.51
MCH (g/dL)	17.28 ± 0.37	17.24 ± 0.15	17.20 ± 0.37	17.22 ± 0.16	17.28 ± 0.28	17.38 ± 0.35	17.20 ± 0.10	17.20 ± 0.07
MCHC (g/dL)	33.28 ± 0.52	33.62 ± 0.50	33.96 ± 0.49	34.16 ± 0.37^a^	34.46 ± 0.25	34.44 ± 0.72	34.36 ± 0.17	34.46 ± 0.39
RETA (× 10^9^/L)	260.70 ± 41.33	241.48 ± 22.83	190.72 ± 17.52	30.48 ± 2.94^d^	186.50 ± 40.82	162.16 ± 28.66	139.66 ± 14.54	137.66 ± 15.18
RET (%)	3.12 ± 0.58	2.79 ± 0.29	2.12 ± 0.20	0.32 ± 0.03^d^	2.07 ± 0.50	1.84 ± 0.33	1.52 ± 0.18	1.44 ± 0.12c
PLT (×10^3^/μL)	928 ± 39	892 ± 23	823 ± 43^b^	510 ± 39^b^	840 ± 49	806 ± 47	844 ± 78	842 ± 73
LYMA (×10^3^/μL)	3.22 ± 0.51	3.58 ± 0.41	2.45 ± 0.58	2.23 ± 0.67^a^	3.35 ± 0.51	3.22 ± 0.58	2.68 ± 0.64	1.78 ± 0.19^b^
MONA (×10^3^/μL)	0.08 ± 0.02	0.10 ± 0.01	0.07 ± 0.03	0.07 ± 0.04	0.13 ± 0.03	0.09 ± 0.01	0.09 ± 0.04	0.07 ± 0.02^b^
EOSA (×10^3^/μL)	0.05 ± 0.01	0.07 ± 0.02	0.08 ± 0.02^a^	0.05 ± 0.02	0.06 ± 0.01	0.07 ± 0.03	0.07 ± 0.02	0.06 ± 0.01
EOS% (%)	1.20 ± 0.27	1.46 ± 0.26	2.34 ± 0.23^a^	1.80 ± 0.90	1.40 ± 0.25	1.78 ± 0.90	2.02 ± 0.31	2.38 ± 0.30
BAS%(%)	0.32 ± 0.05	0.26 ± 0.09	0.18 ± 0.08^a^	0.18 ± 0.08^a^	0.30 ± 0.14	0.32 ± 0.05	0.22 ± 0.08	0.26 ± 0.18
APTT (sec)	16.26 ± 0.67	17.10 ± 0.55	18.12 ± 0.66	21.08 ± 1.89^d^	17.44 ± 1.46	16.88 ± 0.51	17.78 ± 0.70	19.32 ± 0.92^a^
PT (sec)	11.00 ± 0.38	10.98 ± 0.61	12.04 ± 0.42^a^	12.78 ± 0.68^b^	11.18 ± 0.37	11.58 ± 0.30	12.64 ± 0.54^b^	13.70 ± 0.41^b^
Clinical chemistry								
ALT (IU/L)	36.06 ± 3.04	37.28 ± 3.52	35.24 ± 2.64	47.22 ± 11.48^a^	30.14 ± 2.59	28.02 ± 3.39	32.34 ± 3.24	36.52 ± 5.64
ALP (IU/L)	914.28 ± 71.49	896.70 ± 51.47	788.74 ± 45.07^a^	675.46 ± 74.70^b^	599.54 ± 44.44	592.44 ± 52.97	647.14 ± 21.79	639.04 ± 74.65
TG (mg/dL)	53.98 ± 20.09	49.26 ± 9.06	27.02 ± 6.12	17.92 ± 1.44^b^	16.76 ± 3.52	16.26 ± 5.62	12.88 ± 2.39	16.56 ± 2.96
CK (U/L)	618.66 ± 142.76	583.08 ± 163.15	545.66 ± 130.72	244.94 ± 41.77^b^	242.06 ± 35.26	297.80 ± 148.68	257.30 ± 106.39	212.24 ± 63.77
Cl (mmol/L)	94.62 ± 0.71	96.32 ± 3.05	95.56 ± 0.20	91.06 ± 2.88	97.62 ± 1.40	97.78 ± 0.61	99.04 ± 0.63	97.32 ± 1.15
K (mmol/L)	4.52 ± 0.22	4.62 ± 0.13	4.80 ± 0.19	4.64 ± 0.37	4.08 ± 0.16	4.06 ± 0.11	4.32 ± 0.15^a^	4.26 ± 0.13
Na (mmol/L)	134.70 ± 0.49	135.72 ± 2.96	134.98 ± 0.67	136.34 ± 1.176	134.58 ± 0.52	135.22 ± 0.61	136.80 ± 0.83^b^	138.10 ± 0.77^b^

The values are expressed as mean ± SD (*n* = 5 males and 5 females per group)

^a^Dunnett Test Significant at the 0.05 level, ^b^Dunnett Test Significant at the 0.01 level

^c^Dunn Rank Sum Test Significant at the 0.05 level, ^d^Dunn Rank Sum Test Significant at the 0.01 level

**Table 2:** Hematologic and blood chemical parameters of recovery group male rats exposed to benzalkonium chloride

	Recovery group							
Recovery period	2 Weeks				4 Weeks			
Concentration (mg/m^3^)	0	0.8	4	20	0	0.8	4	20
Hematology								
WBC (×10^3^/μL)	4.65 ± 1.10	4.65 ± 0.50	4.48 ± 0.92	4.64 ± 0.71	4.70 ± 0.46	5.38 ± 0.69	4.45 ± 0.54	3.74 ± 1.15
RBC (×10^6^/μL)	9.02 ± 0.31	8.98 ± 0.112	8.96 ± 0.27	8.95 ± 0.16	8.89 ± 0.10	9.00 ± 0.19	8.89 ± 0.16	8.77 ± 0.31
HCT (%)	43.68 ± 1.37	43.40 ± 0.68	43.66 ± 0.84	44.22 ± 0.93	42.16 ± 0.26	42.62 ± 0.66	42.52 ± 0.44	42.78 ± 1.71
Hb (g/dL)	14.84 ± 0.47	14.72 ± 0.30	14.72 ± 0.34	14.90 ± 0.29	14.28 ± 0.24	14.46 ± 0.22	14.38 ± 0.15	14.38 ± 0.50
MCV (fL)	48.42 ± 0.36	48.32 ± 0.36	48.80 ± 0.68	49.38 ± 0.21^b^	47.40 ± 0.39	47.32 ± 0.40	47.84 ± 0.54	48.74 ± 0.55^b^
MCH (g/dL)	16.4 ± 0.1	16.4 ± 0.2	16.4 ± 0.2	16.7 ± 0.1	16.00 ± 0.09	16.10 ± 0.11	16.10 ± 0.25	16.40 ± 0.07^b^
MCHC (g/dL)	33.91 ± 0.07	33.94 ± 0.25	33.70 ± 0.30	33.74 ± 0.30	33.92 ± 0.43	33.96 ± 0.13	33.78 ± 0.31	33.60 ± 0.27
RETA (×10^9^/L)	250.48 ± 34.86	256.74 ± 5.45	328.56 ± 47.76^b^	319.96 ± 30.79^a^	270.34 ± 16.42	239.50 ± 18.57	280.40 ± 25.11	292.90 ± 20.89
RET (%)	2.78 ± 0.40	2.86 ± 0.05	3.67 ± 0.58^b^	3.58 ± 0.33^a^	3.04 ± 0.18	2.66 ± 0.25	3.15 ± 0.32	3.34 ± 0.18
PLT (×10^3^/μL)	788.0 ± 37.29	816.4 ± 11.78	871.4 ± 32.55^c^	872.2 ± 21.35^c^	787 ± 20	773 ± 43	806 ± 36	789 ± 49
LYMA (×10^3^/μL)	3.23 ± 0.87	3.37 ± 0.37	3.13 ± 0.84	3.29 ± 0.42	3.40 ± 0.44	3.89 ± 0.27	2.89 ± 0.72	2.58 ± 0.87
MONA (×10^3^/μL)	0.12 ± 0.05	0.10 ± 0.37	0.11 ± 0.03	0.13 ± 0.05	0.11 ± 0.03	0.12 ± 0.02	0.12 ± 0.02	0.08 ± 0.01
EOSA (×10^3^/μL)	0.07 ± 0.01	0.07 ± 0.01	0.06 ± 0.01	0.07 ± 0.02	0.07 ± 0.03	0.08 ± 0.02	0.06 ± 0.02	0.06 ± 0.01
EOS% (%)	1.44 ± 0.21	1.52 ± 0.26	1.28 ± 0.30	1.48 ± 0.16	1.48 ± 0.48	1.48 ± 0.31	1.42 ± 0.43	1.68 ± 0.31
APTT (sec)	19.48 ± 2.32	18.92 ± 0.66	19.12 ± 0.35	21.04 ± 3.60	16.54 ± 0.68	17.22 ± 0.42	16.70 ± 0.44	16.90 ± 0.57
APT (sec)	10.28 ± 0.16	10.72 ± 0.52	9.98 ± 0.11	10.24 ± 0.24	10.46 ± 0.35	10.68 ± 0.26	10.38 ± 0.42	11.42 ± 0.46^b^
Clinical chemistry								
ALT (IU/L)	49.40 ± 23.18	39.28 ± 2.88	40.52 ± 1.10	42.12 ± 2.80	41.40 ± 1.05	43.18 ± 1.00	42.18 ± 1.25	45.02 ± 6.36
ALP (IU/L)	695.52 ± 28.52	688.96 ± 29.80	777.70 ± 51.71^a^	770.64 ± 54.58^a^	594.10 ± 24.24	579.14 ± 23.36	648.06 ± 40.10	680.92 ± 62.32^a^
TG (mg/dL)	74.30 ± 9.57	66.82 ± 12.77	74.64 ± 24.61	63.28 ± 9.17	104.52 ± 25.24	84.08 ± 27.14	94.68 ± 37.22	59.40 ± 18.83
CK (U/L)	311.82 ± 119.04	270.86 ± 127.51	298.22 ± 125.41	290.66 ± 107.74	453.02 ± 114.84	400.54 ± 191.10	384.44 ± 151.55	435.16 ± 225.82
Cl (mmol/L)	102.48 ± 1.21	103.94 ± 0.85	104.10 ± 0.88	103.24 ± 1.44	102.24 ± 1.30	103.20 ± 1.39	103.96 ± 0.40	104.20 ± 0.91^a^
K (mmol/L)	4.56 ± 0.68	4.36 ± 0.53	4.34 ± 0.57	4.92 ± 1.06	4.40 ± 0.46	4.52 ± 0.80	5.04 ± 0.42	4.56 ± 0.80
Na (mmol/L)	143.74 ± 1.44	144.24 ± 0.81	144.92 ± 0.41	143.92 ± 1.43	143.40 ± 0.68	143.50 ± 0.62	144.34 ± 0.44^a^	144.64 ± 0.49^b^

The values are expressed as mean ± SD (*n* = 5 males per group)

^a^Dunnett Test Significant at the 0.05 level, ^b^Dunnett Test Significant at the 0.01 level

^c^Dunn Rank Sum Test Significant at the 0.01 level

In male rats of the main groups, the results of the blood biochemical analysis showed a statistically significant decrease compare to control in the ALP and CK activities, and TG level and a statistically significant increase in the ALT activity in the T3 group rats. In the T2 group, a statistically significant decrease in the ALP activity was observed, but the remaining parameters showed no statistically significant change compare to control. In female rats of the main groups, the Na level in the T2 and T3 group rats and K level in the T2 group rats showed a statistically significant increase compared with those in the control group. The remaining parameters showed no statistically significant change (Table 1). After 2 weeks of recovery period, a statistically significant increase was observed in the ALP activity in the T3 and T2 male recovery groups. After 4 weeks of recovery period, a statistically significant increase was observed in the Na and Cl levels and ALP activity in the T3 group rats and a statistically significant increase in the Na level in the T2 group rats compare with those in the control (Table 2), but the remaining parameters did not show any statistically significant changes (Additional file 1: Tables S3 and Table S4).

### Organ weights

In the male rats of the T3 group, the absolute weight of brain, heart, lung, liver, spleen and kidney and the relative weight of liver and spleen were statistically significant decreased, and the relative weight of brain, heart, lung and kidney was statistically significant increased compared with those of the control group. In the male rats of the T2 group, the absolute weight of liver, spleen and kidney and the relative weight of liver and spleen were statistically significant decreased, and the relative weight of lung was statistically significant increased compared with those of the control group. In the female rats of the T3 group, the absolute weight of heart, liver, spleen and kidney and the relative weight of spleen were statistically significant decreased, and the relative weight of brain, heart and lung was statistically significant increased compared with those of the control group. In the female rats of the T2 group, the absolute weight of liver and spleen was statistically significant decreased compared with those of the control group (Table 3).

**Table 3:** Absolute and relative organ weights of main group rats exposed to benzalkonium chloride

	Main group							
Sex	Male				Female			
Concentration (mg/m^3^)	0	0.8	4	20	0	0.8	4	20
Absolute organ weight (g)								
Brain	1.767 ± 0.017	1.758 ± 0.041	1.706 ± 0.039^a^	1.683 ± 0.030^b^	1.668 ± 0.020	1.659 ± 0.056	1.624 ± 0.097	1.630 ± 0.056
Heart	0.646 ± 0.026	0.603 ± 0.054	0.536 ± 0.022	0.467 ± 0.032##	0.474 ± 0.034	0.445 ± 0.010	0.435 ± 0.036	0.422 ± 0.022^a^
Lung	0.330 ± 0.031	0.350 ± 0.026	0.349 ± 0.016	0.309 ± 0.022	0.273 ± 0.050	0.286 ± 0.015	0.299 ± 0.016	0.329 ± 0.071
Liver	5.803 ± 0.257	5.282 ± 0.479	4.513 ± 0.476^b^	3.369 ± 0.237^b^	3.664 ± 0.300	3.493 ± 0.102	3.192 ± 0.274^a^	3.082 ± 0.194^b^
Spleen	0.457 ± 0.037	0.438 ± 0.034	0.330 ± 0.021^b^	0.214 ± 0.026^b^	0.329 ± 0.046	0.317 ± 0.023	0.274 ± 0.016^a^	0.220 ± 0.010^b^
Kidney	1.384 ± 0.074	1.319 ± 0.077	1.180 ± 0.082^b^	1.031 ± 0.068^b^	0.999 ± 0.068	0.978 ± 0.038	0.926 ± 0.051	0.895 ± 0.066^a^
Relative organ weight (%)								
Brain	0.934 ± 0.049	0.978 ± 0.078	1.083 ± 0.107	1.383 ± 0.119^b^	1.274 ± 0.064	1.309 ± 0.073	1.359 ± 0.054	1.507 ± 0.073^b^
Heart	0.342 ± 0.021	0.333 ± 0.009	0.340 ± 0.024	0.382 ± 0.015^b^	0.362 ± 0.017	0.351 ± 0.011	0.364 ± 0.016	0.390 ± 0.020^a^
Lung	0.175 ± 0.022	0.194 ± 0.010	0.221 ± 0.016^b^	0.254 ± 0.020^b^	0.208 ± 0.033	0.226 ± 0.009	0.251 ± 0.031	0.304 ± 0.066^b^
Liver	3.064 ± 0.091	2.923 ± 0.110	2.841 ± 0.085^b^	2.757 ± 0.112^b^	2.791 ± 0.011	2.753 ± 0.077	2.667 ± 0.115	2.844 ± 0.084
Spleen	0.241 ± 0.023	0.242 ± 0.009	0.208 ± 0.009^b^	0.175 ± 0.014^b^	0.250 ± 0.025	0.250 ± 0.016	0.230 ± 0.021	0.204 ± 0.011^b^
Kidney	0.732 ± 0.054	0.732 ± 0.049	0.745 ± 0.024	0.843 ± 0.023^b^	0.761 ± 0.021	0.771 ± 0.042	0.775 ± 0.050	0.826 ± 0.048

The values are expressed as mean ± SD (*n* = 5 males and 5 females per group)

^a^Dunnett Test Significant at the 0.05 level, ^b^Dunnett Test Significant at the 0.01 level

##Dunn Rank Sum Test Significant at the 0.01 level

In the male rats of the recovery group, the relative weight of the brain, heart, and lung of the T3 group was statistically significant increased, and the absolute weight of the liver was statistically significant decreased compared with those of the control group after 2 weeks of recovery period. After 4 weeks of recovery period, the relative weight of the brain in the T3 and T2 groups was statistically significant increased compared with that in the control group (Table 4).

**Table 4:** Absolute and relative organ weights of recovery group male rats exposed to benzalkonium chloride

	Recovery group							
Recovery period	2 Weeks				4 Weeks			
Concentration (mg/m^3^)	0	0.8	4	20	0	0.8	4	20
Absolute organ weight (g)								
Brain	1.751 ± 0.101	1.804 ± 0.053	1.796 ± 0.018	1.695 ± 0.081	1.831 ± 0.061	1.774 ± 0.095	1.815 ± 0.030	1.786 ± 0.100
Heart	0.702 ± 0.076	0.718 ± 0.030	0.700 ± 0.037	0.681 ± 0.032	0.771 ± 0.034	0.743 ± 0.037	0.736 ± 0.061	0.721 ± 0.086
Lung	0.387 ± 0.035	0.424 ± 0.023	0.394 ± 0.035	0.388 ± 0.021	0.430 ± 0.030	0.419 ± 0.030	0.438 ± 0.043	0.416 ± 0.012
Liver	6.765 ± 0.493	6.738 ± 0.403	6.512 ± 0.131	5.847 ± 0.140^b^	7.545 ± 0.576	7.225 ± 0.697	6.987 ± 0.605	6.785 ± 0.578
Spleen	0.517 ± 0.031	0.547 ± 0.027	0.534 ± 0.066	0.488 ± 0.024	0.621 ± 0.064	0.582 ± 0.039	0.575 ± 0.058	0.564 ± 0.036
Kidney	1.573 ± 0.101	1.566 ± 0.100	1.538 ± 0.086	1.424 ± 0.059	1.662 ± 0.122	1.625 ± 0.103	1.583 ± 0.067	1.557 ± 0.070
Relative organ weight (%)								
Brain	0.772 ± 0.053	0.799 ± 0.030	0.826 ± 0.014	0.871 ± 0.059^b^	0.700 ± 0.031	0.707 ± 0.040	0.761 ± 0.021^a^	0.761 ± 0.021^a^
Heart	0.309 ± 0.025	0.318 ± 0.010	0.322 ± 0.016	0.349 ± 0.010^b^	0.295 ± 0.013	0.296 ± 0.014	0.307 ± 0.025	0.307 ± 0.025
Lung	0.171 ± 0.015	0.188 ± 0.016	0.181 ± 0.013	0.199 ± 0.015^a^	0.165 ± 0.019	0.167 ± 0.007	0.177 ± 0.006	0.177 ± 0.006
Liver	2.974 ± 0.082	2.981 ± 0.088	2.992 ± 0.045	3.002 ± 0.054	2.878 ± 0.041	2.870 ± 0.139	2.888 ± 0.126	2.889 ± 0.126
Spleen	0.228 ± 0.009	0.242 ± 0.010	0.245 ± 0.025	0.250 ± 0.008	0.237 ± 0.010	0.232 ± 0.009	0.238 ± 0.012	0.240 ± 0.008
Kidney	0.692 ± 0.018	0.693 ± 0.024	0.706 ± 0.030	0.732 ± 0.032	0.656 ± 0.019	0.647 ± 0.020	0.664 ± 0.017	0.664 ± 0.017

The values are expressed as mean ± SD (*n* = 5 males per group)

^a^Dunnett Test Significant at the 0.05 level, ^b^Dunnett Test Significant at the 0.01 level

### Gross and histopathological findings

In the male rats, black focus of the lungs and a decrease in the size of the liver, spleen, thymus, testis, epididymis, seminal vesicle, and prostate were observed in the T3 group. In the female rats, a decrease in the size of thymus, uterus, and vagina was observed in the T3 group and a decrease in the size of the uterus and vagina was observed in the T2 group. After the recovery period, these necropsy findings in BAC exposed male rats were not observed (data not shown).

**Fig. 4:**
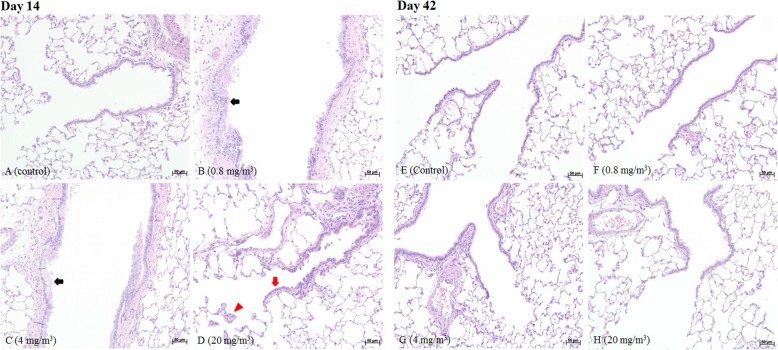
In the lungs of male rats exposed to BAC, black arrows indicate hypertrophy and hyperplasia; red arrows indicate degeneration and regeneration; red triangle indicates smooth muscle hypertrophy

**Fig. 5:**
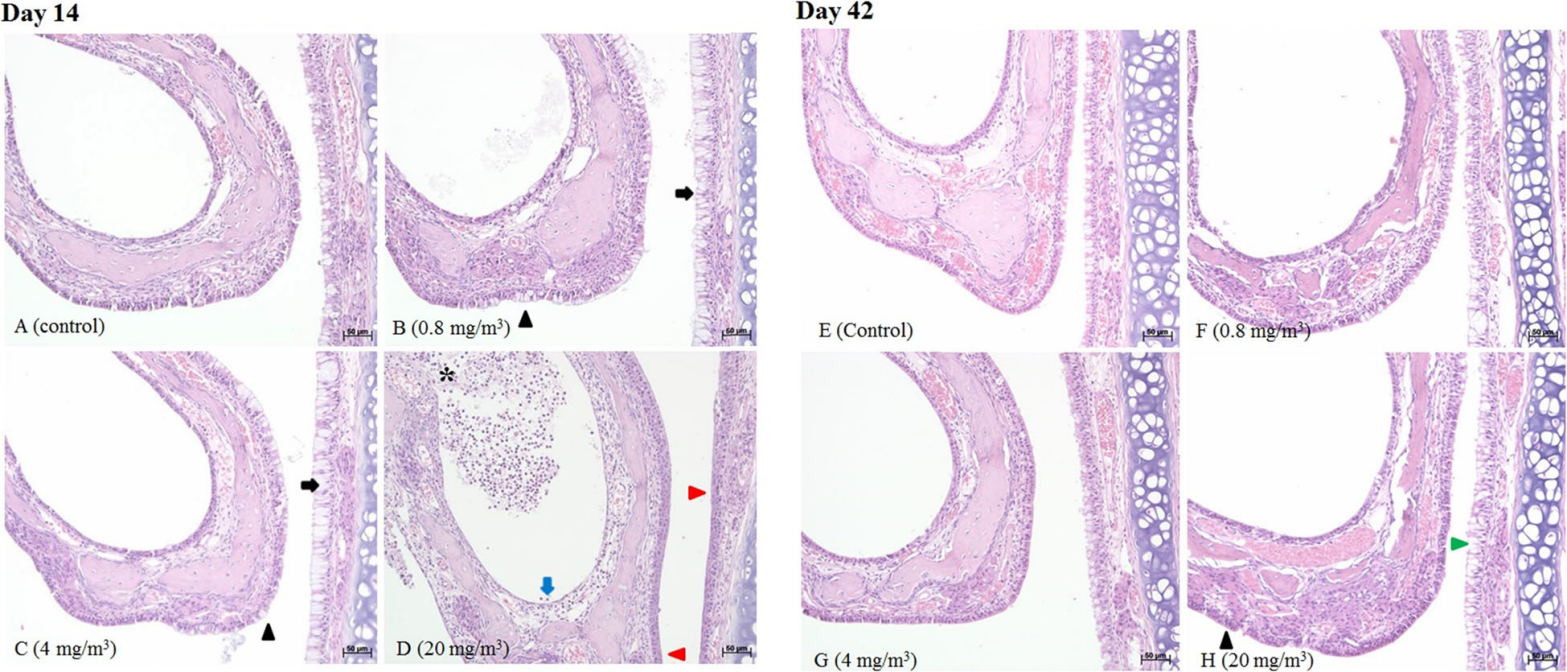
In the nasal tissues of male rats exposed to BAC, black arrows indicate hypertrophy and hyperplasia; black triangles indicate metaplasia; red arrows indicate squamous metaplasia; blue arrows indicate erosion with necrosis; asterisk indicates ulceration with suppurative inflammation; green filled triangles indicate hyperplasia

Histological examinations were conducted to discern the morphological differences between the end of the BAC exposure period and the end of the recovery period. Representative micrographs of the lung and nasal tissues of the control and test groups are shown in Figs. 4–5 and Tables 5–6 [11]. The rats from the control group showed a normal parenchyma at all time points. However, at the end of exposure, degeneration and regeneration of terminal bronchiolar epithelium, smooth muscle hypertrophy of bronchioloalveolar junction, cell debris in the alveolar lumens was observed in the male T2 and T3 groups and female T3 group. Hypertrophy and hyperplasia of mucous cells in the bronchi or bronchiole were observed in both males and females. In the nasal cavity, ulceration with suppurative inflammation, squamous metaplasia, and erosion with necrosis were observed in the respiratory epithelium and transitional epithelium of the male and female T3 groups. Hypertrophy and hyperplasia of mucous cells in the respiratory epithelium were observed in both males and females, and metaplasia of mucous cells in the transitional epithelium was observed in the male and female T1 and T2 groups. In addition, atrophy of olfactory epithelium was observed in the male and female T3 groups.

**Table 5:** Histopathological assessment of the lung and nasal cavity tissues

	Main group							
Sex	Male				Female			
Concentration (mg/m^3^)	0	0.8	4	20	0	0.8	4	20
Lung								
Number of animals	5	5	5	5	5	5	5	5
Degeneration/regeneration, terminal bronchiolar epithelium	(0)	(0)	(2)	(5)	(0)	(0)	(0)	(3)
Minimal	0	0	2	3	0	0	0	3
Mild	0	0	0	2	0	0	0	0
Mean ± SD	0	0	0.40 ± 0.55	1.40 ± 0.55	0	0	0	0.60 ± 0.55
Hypertrophy/hyperplasia, mucous cells, bronchi/bronchiole	(0)	(5)	(5)	(3)	(0)	(3)	(5)	(4)
Minimal	0	0	0	2	0	3	1	3
Mild	0	5	3	1	0	0	2	1
Moderate	0	0	2	0	0	0	2	0
Mean ± SD	0	2.00 ± 0.00	2.40 ± 0.55	0.80 ± 0.84	0	0.60 ± 0.55	2.20 ± 0.84	1.00 ± 0.71
Smooth muscle hypertrophy, bronchioloalveolar junction	(0)	(0)	(3)	(5)	(0)	(0)	(0)	(3)
Minimal	0	0	3	5	0	0	0	3
Mean ± SD	0	0	0.60 ± 0.55	1.00 ± 0.00	0	0	0	0.60 ± 0.55
Infiltration, eosinophil, perivascular	(0)	(1)	(3)	(1)	(0)	(0)	(0)	(2)
Minimal	0	1	3	1	0	0	0	2
Mean ± SD	0	0.20 ± 0.45	0.60 ± 0.55	0.20 ± 0.45	0	0	0	0.40 ± 0.55
Cellular debris, alveolar lumen	(0)	(0)	(3)	(2)	(0)	(0)	(0)	(1)
Minimal	0	0	3	2	0	0	0	1
Mean ± SD	0	0	0.60 ± 0.55	0.40 ± 0.55	0	0	0	0.20 ± 0.45
Nasal cavity								
Number of animals	5	5	5	5	5	5	5	5
Ulceration with suppurative inflammation, respiratory epithelium	(0)	(0)	(0)	(1)	(0)	(0)	(0)	(2)
Mild	0	0	0	1	0	0	0	1
Marked	0	0	0	0	0	0	0	1
Mean ± SD	0	0	0	0.40 ± 0.89	0	0	0	1.20 ± 1.79
Ulceration with suppurative inflammation, transitional epithelium	(0)	(0)	(0)	(3)	(0)	(0)	(0)	(0)
Mild	0	0	0	1	0	0	0	0
Moderate	0	0	0	0	0	0	0	1
Marked	0	0	0	2	0	0	0	0
Mean ± SD	0	0	0	2.00 ± 2.00	0	0	0	0.60 ± 1.34
Squamous metaplasia, respiratory epithelium	(0)	(0)	(0)	(5)	(0)	(0)	(0)	(5)
Minimal	0	0	0	0	0	0	0	1
Mild	0	0	0	2	0	0	0	2
Moderate	0	0	0	3	0	0	0	1
Marked	0	0	0	0	0	0	0	1
Mean ± SD	0	0	0	2.20 ± 1.10	0	0	0	2.40 ± 1.14
Squamous metaplasia, transitional epithelium	(0)	(0)	(0)	(5)	(0)	(0)	(0)	(1)
Minimal	0	0	0	0	0	0	0	4
Mild	0	0	0	4	0	0	0	1
Moderate	0	0	0	1	0	0	0	0
Mean ± SD	0	0	0	1.40 ± 0.89	0	0	0	1.20 ± 0.45
Erosion with necrosis, respiratory epithelium	(0)	(0)	(0)	(5)	(0)	(0)	(0)	(3)
Minimal	0	0	0	3	0	0	0	0
Mild	0	0	0	1	0	0	0	1
Moderate	0	0	0	1	0	0	0	1
Marked	0	0	0	0	0	0	0	1
Mean ± SD	0	0	0	1.60 ± 0.89	0	0	0	1.80 ± 1.79
Erosion with necrosis, transitional epithelium	(0)	(0)	(0)	(5)	(0)	(0)	(0)	(4)
Minimal	0	0	0	2	0	0	0	1
Mild	0	0	0	1	0	0	0	0
Moderate	0	0	0	1	0	0	0	1
Marked	0	0	0	1	0	0	0	2
Mean ± SD	0	0	0	2.20 ± 1.30	0	0	0	2.40 ± 1.82
Hypertrophy/hyperplasia, mucous cells, respiratory epithelium	(0)	(5)	(5)	(5)	(0)	(5)	(5)	(5)
Minimal	0	0	1	1	0	0	1	0
Mild	0	3	0	2	0	0	2	5
Moderate	0	2	4	2	0	4	2	0
Marked	0	0	0	0	0	1	0	0
Mean ± SD	0	2.40 ± 0.55	2.60 ± 0.89	2.20 ± 0.84	0	3.20 ± 0.45	2.20 ± 0.84	1.20 ± 0.45
Metaplasia, mucous cells, transitional epithelium	(0)	(5)	(5)	(0)	(0)	(5)	(5)	(0)
Minimal	0	0	4	0	0	0	1	0
Mild	0	1	1	0	0	1	4	0
Moderate	0	4	0	0	0	4	0	0
Mean ± SD	0	2.80 ± 0.45	1.20 ± 0.45	0	0	2.80 ± 0.45	1.80 ± 0.45	0
Atrophy, olfactory epithelium	(0)	(0)	(0)	(1)	(0)	(0)	(0)	(2)
Mild	0	0	0	1	0	0	0	1
Moderate	0	0	0	0	0	0	0	1
Mean ± SD	0	0	0	0.20 ± 0.45	0	0	0	0.60 ± 0.89

0: unremarkable = no presence of histopathologic lesion; 1: minimal = lesions involving< 10% of the tissue of each organ; 2: mild = lesions involving< 10–30% of the tissue of each organ; 3: moderate = lesions involving< 30–50% of the tissue of each organ; 4: marked = lesions involving< 50–70% of the tissue of each organ; 5: severe = lesions involving> 70% of the tissue of each organ

**Table 6:** Histopathological assessment of the lung and nasal cavity tissues in male rats

	Recovery group							
Recovery period	2 Weeks				4 Weeks			
Concentration (mg/m^3^)	0	0.8	4	20	0	0.8	4	20
Nasal cavity								
Number of animals	5	5	5	5	5	5	5	5
Hyperplasia, transitional epithelium	(0)	(0)	(2)	(5)	(0)	(0)	(0)	(2)
Minimal	0	0	2	0	0	0	0	2
Mild	0	0	0	4	0	0	0	0
Moderate	0	0	0	1	0	0	0	0
Mean ± SD	0	0	0.40 ± 0.55	2.20 ± 0.45	0	0	0	0.40 ± 0.55
Hypertrophy/hyperplasia, mucous cells, respiratory epithelium	(0)	(4)	(5)	(5)	(0)	(0)	(0)	(5)
Minimal	0	4	2	0	0	0	0	4
Mild	0	0	3	2	0	0	0	1
Moderate	0	0	0	3	0	0	0	0
Mean ± SD	0	0.80 ± 0.45	1.60 ± 0.55	2.60 ± 0.55	0	0	0	1.20 ± 0.45
Metaplasia, mucous cells, transitional epithelium	(0)	(4)	(4)	(0)	(0)	(0)	(0)	(0)
Minimal	0	3	1	0	0	0	0	0
Mild	0	1	3	0	0	0	0	0
Mean ± SD	0	1.00 ± 0.71	1.40 ± 0.89	0	0	0	0	0
Infiltration, submucosa, transitional epithelium	(0)	(0)	(0)	(2)	(0)	(0)	(0)	(1)
Minimal	0	0	0	1	0	0	0	1
Mild	0	0	0	1	0	0	0	0
Mean ± SD	0	0	0	0.60 ± 0.89	0	0	0	0.20 ± 0.45
Infiltration, submucosa, respiratory epithelium	(0)	(0)	(0)	(1)	(0)	(0)	(0)	(0)
Mild	0	0	0	1	0	0	0	0
Mean ± SD	0	0	0	0.40 ± 0.89	0	0	0	0
Squamous metaplasia, transitional epithelium	(0)	(0)	(0)	(4)	(0)	(0)	(0)	(1)
Minimal	0	0	0	4	0	0	0	1
Mean ± SD	0	0	0	0.80 ± 0.45	0	0	0	0.20 ± 0.45

0: unremarkable = no presence of histopathologic lesion; 1: minimal = lesions involving< 10% of the tissue of each organ; 2: mild = lesions involving< 10–30% of the tissue of each organ; 3: moderate = lesions involving< 30–50% of the tissue of each organ; 4: marked = lesions involving< 50–70% of the tissue of each organ; 5: severe = lesions involving> 70% of the tissue of each organ

In the nasal cavity, after 2 weeks of recovery period, hyperplasia of transitional epithelium in the T2 and T3 recovery groups, hypertrophy and hyperplasia of mucous cells in respiratory epithelium in all the recovery groups, metaplasia of mucous cells in transitional epithelium in the T1 and T2 recovery groups, infiltration of submucosa in transitional and respiratory epithelium in the T3 recovery group, and squamous metaplasia in transitional epithelium in the T3 recovery group were observed. After 4 weeks of recovery period, hyperplasia in transitional epithelium, hypertrophy and hyperplasia of mucous cells in respiratory epithelium, infiltration of submucosa, and squamous metaplasia in transitional epithelium were observed in the T3 recovery group.

### Analysis of BALF

**Fig. 6:**
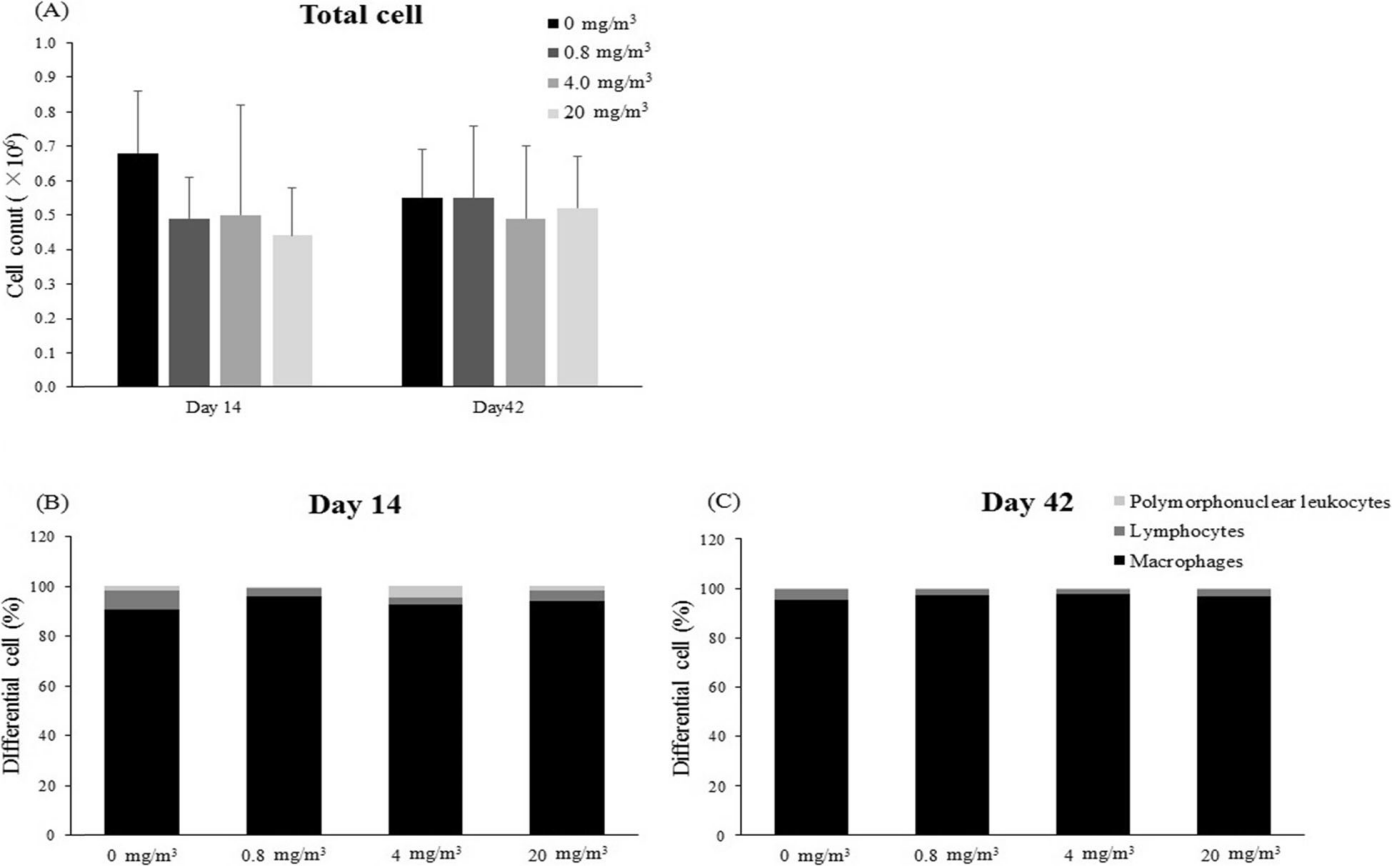
Total cell counts from bronchoalveolar lavage fluid (BALF) and composition of cell population as a percentage of total cells after BAC exposure (**a**-**c**). The values are expressed as mean ± SD (*n* = 5 males per group)

**Fig. 7:**
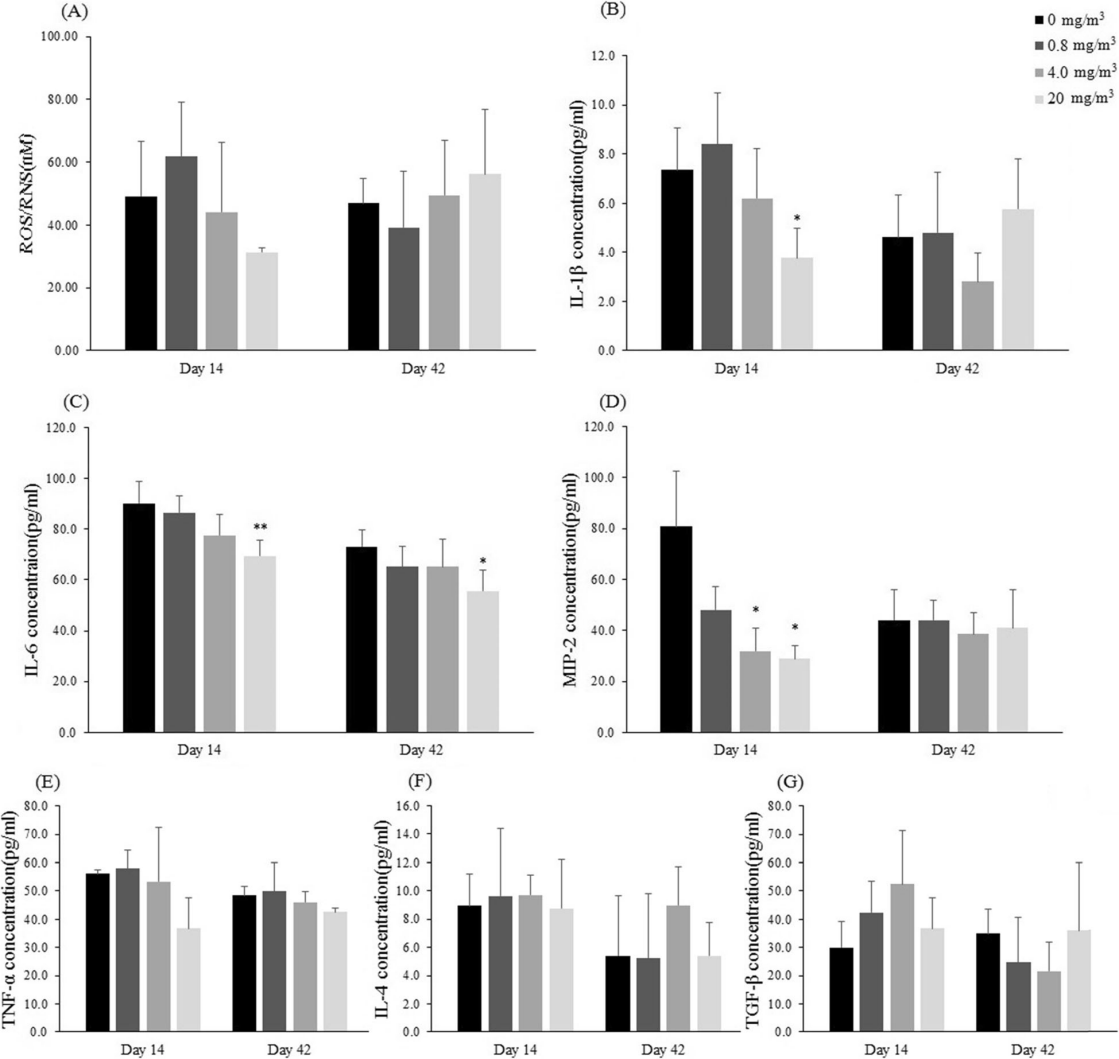
Concentrations of cytokines in bronchoalveolar lavage fluid (**a**-**g**). The values are expressed as mean ± SD (*n* = 5 males per group). Significantly different from control by Dunn Rank Sum test: *p < 0.05, **p < 0.001

There was no statistically significant difference in the total cell and differential cell counts between the BALF obtained at the end of exposure period (14 days) and 4 weeks of recovery (42 days) (Fig. 6). We measured the concentration of reactive oxygen species (ROS) and reactive nitrogen species (RNS), as an index of oxidative damage, and cytokines (IL-1β, TNF-α, IL-4, IL-6, MIP-2, and TGF-β) in the BALF. The concentrations of IL-1β, IL-6 and MIP-2 showed a statistically significant decrease at the end of the exposure period, but these changes were not observed in the 4-week recovery group except IL-6 in the T3 group (Fig. 7). In addition, the concentration of ROS/RNS showed a concentration-dependent decrease, although not a statistically significant change. However, the concentrations of TNF-α, IL-4 and TGF-β did not show statistically significant changes after exposure to the test substance.

## Discussion

Benzalkonium chloride, a quaternary ammonium compound, is a mixture of several n-alkylbenzyldimethylammonium chlorides (*n* = 10–16) with different alkyl groups [12]. It is a cationic surfactant used as a bactericide or preservative owing to its inhibitory action against bacteria and fungi [13]. It is known that the long alkyl group of BAC interferes with the double-layered bacterial cell membrane, destroys it, and leaks the cell contents, and thus inhibits bacterial growth [14, 15]. Studies have reported the side effects of BAC, such as skin irritation and dermatitis due to exposure via inhalation [16–18].

Globally, BAC is commonly used as sterilizing and preserving agents in household chemical products such as spray-type antimicrobial agents, perfumes [19], and deodorants. Some studies have reported respiratory toxicity of BAC. Exposure to BAC induced cytotoxicity and DNA damage in human bronchial cell line (BEAS-2B) [20], and acute or repeated inhalation of BAC induced lung irritation, inflammation, and alveolar damage [3, 21, 22]. In addition, orally or intravenously administered BAC has been reported to accumulate predominantly in the lungs of rats, leading to pulmonary edema and pneumonia [23, 24]. Therefore, the lungs are considered to be the main target organ of BAC. Recently, BAC is suspected to be one of the causative substances of toxicity involving humidifier fungicides in Korea. In the present study, the inhalation toxicity test was performed to investigate the toxicity of BAC inhalation.

To evaluate the toxic effects of repeated exposure to BAC, F344 rats were exposed to 0.8, 4, and 20 mg/m^3^ BAC, 6 h/day for 14 days. We also set up a recovery period of two and 4 weeks to assess the reversibility of these effects. The mean concentration of BAC in the whole-body exposure chamber measured during the exposure period was in accordance with that used in the inhalation test for aerosols because the error from the target concentration was ≤20%. The MMAD and GSD of the aerosols measured during the exposure period also met the aerosol inhalation test requirements in the OECD TG412 [25].

During the exposure period, nasal discharge, rale, and deep respiration were observed in the T3 group, and nasal discharge observed in the T1 and T2 groups. These clinical signs are thought to be an emergency airway response caused by the irritation of BAC. The *emergency airway defense response* consists of glottal closure, airway constriction, pulmonary vessel dilation, cough, and copious gland secretion. The emergency airway defense response is centrally mediated and depends on intact vagal connections to the lungs [26]. During the exposure period, a significant weight loss was observed in males in the T2 and T3 groups and females in the T3 test group, and more weight loss was observed in males than in females, but both sexes showed similar changes in weight. The body weight loss was accompanied by the decreased feed intake. These reductions were considered related to the exposure to the test substance.

The hematological test results showed changes in the RBC count, HCT level, HGB level, and MCHC in the male T3 group rats. The results also showed changes in the RBC count, and HCT and HGB levels in the male T2 group rats and the HGB level in the male T1 group rats. These changes are considered to be due to weight loss with dehydration [27].

The changes in the MCV, reticulocyte count and ratio, PLT count, APTT, and PT in the male T3 group rats; the reticulocyte count and ratio, PLT count, and PT in the male rats exposed to T2 group rats; the reticulocyte count and ratio, APTT, and PT in the female T3 group rats; and the PT in the female T2 group rats were observed. These changes can be attributed to the reduction in hematopoietic function due to the decrease in feed intake and the subsequent weight loss [28, 29]. The changes in the number of lymphocytes observed in the T3 group rats were inferred to be due to nutritional deficiency or stress due to weight loss [30]. In addition, there was no toxicological significance in the basophil count of the male T2 and T3 group rats and in the monocyte count of the female T3 group rats.

In the blood biochemical test, the increase in the ALT activity in the male T3 group rats is considered to be related to liver atrophy observed in histopathological examination [28] (Additional file 1: Table S5). On the contrary, the changes in the ALP activity in the male T3 group rats and the TG level and CK activity in the male T3 group rats were considered to be due to the decrease in feed intake causing weight loss. In addition, the significant increase in the K and Na levels observed in female rats is thought to be due to dehydration by epithelial cell stimulation by BAC.

In the BALF analysis, the concentration of ROS/RNS, IL-1β, IL-6, and MIP-2 decreased dose dependently at the end of the exposure period, but did not show a concentration-dependent change at 4 weeks of recovery. In addition, the concentrations of TNF-α, IL-4, and TGF-β did not show changes associated with test substance exposure. Although IL-6 is traditionally considered as proinflammatory cytokine, it is highly polymorphic and its anti-inflammatory activity has been reported [31]. In addition, a reduction in inflammatory cells, decrease in chemokine and inflammatory cytokine expression, and alteration in macrophage mobilization in JP-8-induced dermatitis strongly suggest the role of anti-inflammatory response, rather than inducing IL-6 inflammatory response. These changes in the expression or function of these cytokines might modulate stimulatory sensitivity in human [32]. The concentration-dependent decrease in the concentrations of ROS/RNS, IL-1β, IL-6, and MIP-2 in this study was caused by the stimulation of the test substance, and cytokines such as IL-6, IL-1β, and MIP-2 are thought to function together.

In the measurement of organ weights, changes in the weights of the brain, heart, lungs, liver, spleen, and kidneys of the male and female T2 and T3 group rats, size reduction and atrophy of the liver and spleen in the male and female T3 group rats (Additional file 1: Table S5) were considered to be due to weight loss [33, 34]. In the recovery group, the body and organ weights of rats gradually improved.

The autopsy results showed that the black lesions in the lungs of male T3 group rats were consistent with the hemoglobin crystallographic findings of the alveolar muscle in the histopathological examination. The size reduction observed in the thymus, testis, epididymis, seminal vesicles, prostate, uterus, and vagina was consistent with the atrophic findings in the macroscopic examination. Furthermore, the squamous metaplasia of the respiratory epithelium and transitional epithelium, mucinous cell hypertrophy and proliferation of the respiratory epithelium, mucinous cell metaplasia of the transitional epithelium in the nasal cavities, and mucinous cell hypertrophy and proliferation of terminal bronchiole are considered adaptive changes after tissue injury [35]. The atrophy of the thymus was considered to be due to stress from weight loss [36].

In histopathological examination, changes in the respiratory epithelium and transition epithelium of the nasal cavity, atrophy of the erosive and olfactory epithelium with necrosis, denaturation and regeneration of the respiratory bronchial epithelium, hypertrophy of the smooth muscle in the bronchial alveolar junction, and cell debris indicated damage due to stimulation by the test substance. Peripheral eosinophil infiltration in the lung is thought to be an allergic reaction because it is associated with increased eosinophil ratio and eosinophil count.

Histopathological findings in the nasal cavity and lungs verified that BAC induced irritation in the nasal cavity and the lungs, which were the main organs affected in the respiratory system. ROS/RNS, IL-1β, IL-6, and MIP-2 levels decreased in a BAC concentration-dependent manner, indicating that BAC exposure caused oxidative damage. The decrease in the level of IL-6, an anti-inflammatory agent, led to a decrease in ROS/RNS, an indicator of oxidative damage. At this time, IL-1β and MIP-2, together with IL-6, are considered to be acting as cytokines. Therefore, additional research is needed to clarify this hypothesis.

Inhaled substances may affect the respiratory system at various levels according to various factors, such as the characteristics of substances, environment, and host factors. The nasal cavity is important in inhalation toxicology because nose is the first part of the respiratory tract that contacts and filters airborne particles [37]. The mucosa in the nasal cavity is the first tissue of defense against inhaled particles in upper airway [38, 39]. Inhaled particles are trapped in mucus and removed to be swallowed by the coordination with the movement of ciliary epithelium [38, 39]. BAC has been shown to inhibit the nasal mucociliary activities via damaging the ciliated nasal epithelial cells [40, 41, 42]. BAC is a human skin and severe eye irritant [43]. It is a suspected respiratory toxicant, immunotoxicant, gastrointestinal toxicant, and neurotoxicant [44–46]. BAC for consumer use are dilute solutions. Concentrated solutions are toxic to humans, causing corrosion/irritation to the skin and mucosa, and death if taken internally in sufficient volumes. Several studies have shown the nasal toxicity of BAC in animals and humans. The administration of 0.05 and 0.10% (*w*/*v*) BAC solutions to the nasal cavity of rats caused epithelial inflammation, desquamation, and edema in the dorsal meatus and adjacent nasal septum [47]. BAC-containing nasal decongestant sprays induced or aggravated nasal swelling and stuffiness in healthy volunteers and patients with rhinitis compared with those of sprays without BAC [48–50]. Recently, the adverse effects of BAC through inhalation exposure and the target organ of BAC toxicity can be shifted to the upper respiratory organs rather than the deeper lower airway [51]. In this study, more exposure-related effects were observed in the upper airway. As mentioned above, BAC is thought to be more exposed to the upper respiratory tract due to mucociliary clearance and emergency airway response caused by the irritation of BAC. In addition, NOAEL is considered to be less than 0.8 mg/m^3^ because the effects associated with BAC exposure were also observed in the nasal cavity of rats exposed to a concentration of mg/m^3^.

Thus, from these results, we calculated the BMD value because the exposure criteria are required to protect the health of workers handling BAC. The BMD recommended by the Environmental Protection Agency should be calculated to overcome the drawbacks of NOAEL, which is dependent on exposure concentration. The BMD calculation yielded the BMDL, the 95% lower confidence of the dose corresponding to 10% reaction incidence, and the lowest value was selected as the BMD value. The toxicity data for dose response were body weight, lung weight, RBC count, HCT level, Hb level, MCHC, MCV, RETA, RET%, PLT count, LYMA, APTT, PT, TG level, and ALT, ALP, and CK activities, and the models were Exponential and Hill. The BMDL values obtained were 0.10, 0.0031, 0.63, 1.53, 1, 37.5, 33.5, 0.55, 0.39, 0.89, 0.29, 0.12, 0.26, 0.86, 0.085, 0.082, and 0.208 mg/m^3^, respectively. Therefore, we chose 0.0031 mg/m^3^ as the BMD value, the dose corresponding to lung weight-related dose-response, and the DNEL was 0.000062 mg/m^3^, determined by applying an interspecies factor of 2.5, intraspecies factor of 5, and exposure duration factor of 4 as default assessment factors [52].

## Conclusions

BAC could be introduced to the occupational environment of the workers handling it, and it was necessary to identify the hazard as a causative substance of the humidifier disinfectant accident. Because biocides are widely used, the risk of accidental exposure is high and consequently, there is a need to curb the health problems due to such exposures in workers in various fields. Therefore, this study was conducted to evaluate the effects of BAC inhalation exposure for 14 days using an inhalation chamber system and a mist generator. Overall, the study results confirmed that the main targets of repetitive systemic inhalation of BAC are the respiratory system, including the nasal cavity and lungs. In particular, BAC was found to cause irritation in the nasal cavity and lungs, which are located in a relatively upper position than the deep lower airway of the respiratory system, and the irritation was caused by oxidative damage; it is thought that IL-6, IL-1β, and MIP-2 cytokines functioned together. In addition, the parasympathetic nerve responded to the animal’s defensive response to these irritations, resulting in clinical signs of nasal discharge, rale, and deep respiration. It is judged that the animals’ health is worsened as a result of weight loss due to reduced feed intake. These changes led to changes in blood biochemistry and hematological parameters, which gradually recovered during the recovery period. From the results, the NOAEL was considered to be less than 0.8 mg/m^3^. Therefore, by applying the dose-response data using a mathematical model, the BMD was calculated to be 0.0031 mg/m^3^. The DNEL value converted from these BMD values to human exposure according to information requirements and chemical safety assessment guidelines was defined as 0.000062 mg/m^3^.

## Methods

### Chemicals and animals

Benzalkonium chloride (concentration 50.5%) was purchased from Samchun Chemicals (Pyeongtaek, Korea). To generate the aerosol form of BAC in the whole-body exposure chamber, BAC was diluted to 1–2% (v/v) with microfiltration and by using UV-sterilized water.

Six-week-old Fischer 344 rats were supplied by Japan SLC Inc. (Shizuoka, Japan) and were used in the experiments after an 8-day acclimation period. The body weight range of males at the start of exposure to BAC after the acclimation period was 129.40–190.07 g and that of females was 122.07–145.58 g. The rats were individually housed in six-wire mesh cages (W 240 mm × L 1200 mm × H 200 mm) during the exposure period. The environment conditions were as follows: temperature, 22 °C ± 3 °C; relative humidity, 30–70%; light/dark cycle, 12 h each; light intensity, 150–300 Lux; and air ventilation, 10–15 times/h. The rats were fed pelleted food (ENVIGO RMS Inc., Indianapolis, IN, USA) sterilized using gamma rays and were provided filtered and sterilized tap water ad libitum. All animal experiments were approved by the Institute Animal Care and Use Committee of Occupational Safety and Health Research Institute (AEC-200806230002).

### Experimental design

Based on their body weight, the rats were randomly allocated to five animals per test group, using PRISTIMA 7.1.0 software (Xybion Medical Systems Corporation, Morris Plains, NJ, USA). The male and female rats were divided into the following four main groups: a control and three test groups; the test group rats were subjected to whole body exposure to BAC for 14 days. In addition, to assess the effects of cytokines on oxidative damage and toxic reversibility, only male rats were provided the recovery periods of 2 and 4 weeks, respectively. The experimental concentrations of BAC were selected based on the results of our previous acute inhalation toxicity study. In the acute inhalation toxicity study of BAC conducted in accordance with OECD TG 436, bleeding from the nasal cavity was observed in all the rats after exposure to 50 mg/m^3^ BAC. Therefore, in this study, the highest concentration of 20 mg/m^3^ (T3 group), which is expected to induce no mortality and repeated toxicity upon repeated inhalation exposure, was set as the high concentration of exposure, and 4 (T2 group) and 0.8 mg/m^3^ (T1 group) were set as the medium and low concentrations, respectively, using a common ratio 5. In Korea, when BAC is used as a humidifier disinfectant, the concentration is 1.869 mg/m^3^ (product concentration 0.045%, 2.5 g of product is diluted in 500 mL of water; the maximum spray volume of humidifier 500 mL/h, 24-h use, average volume of room 30.3 m^3^, and winter air change rate 0.2/h) [53]. Thus, the low concentration set in this study was 0.43 times the actual environmental concentration. The control group was exposed to clean air passed through high efficiency particulate air filters.

### Exposure and monitoring

The prepared test substance was aerosolized using an atomizer-type mist generator (NB-2 N; Sibata Co. Ltd., Japan). Diluted air obtained using the Aerosol Dilution System was mixed with the generated aerosol and supplied to the whole-body exposure chamber (1.4 m^3^). The control group was supplied only clean air without the test substance and the housing environment condition was the same as that of the test groups.

To confirm the concentration of the test substance in the chambers, samples of the test substance were collected three times from the respirable area of the rats in the chambers using a personal sample collector (Airchek® XR 5000 Sample Pump, SKC Inc., PA 15330, USA) connected to a filter holder with a 25-mm micro glass fiber filter. The actual concentrations in the chambers were determined using the gravimetric method to calculate the filter weight measured before and after sampling. In addition, the number of aerosol particles in the chamber was checked using a Portable Aerosol Spectrometer (GRIMM Aerosol Ainring Technik GmbH & Co. KG, Salzburg, Germany) in real time during the generation of test substance. The mass median aerodynamic diameter (MMAD) and geometric standard deviation (GSD) for each exposure concentration were determined once during the exposure period using a Cascade impactor (MiniMOUDI Impactor, MSP Co. Ltd., Minnesota 55,126, USA).

### Observation and measurements

Clinical signs were observed daily during the experiment and the body weight of the rats was measured on the day of exposure, twice a week during the exposure period, once a week during the recovery period, and on the day of autopsy. Feed intake by the rats was measured once a week during the exposure and recovery periods. At the time of autopsy, blood was collected from all the rats under the influence of isoflurane inhalation anesthesia. The rats were then euthanised by bleeding. The external surface, all orifices, and the organs of the abdominal, thoracic, and cranial cavities were examined. Subsequently, the brain, liver, heart, lungs, spleen, and kidneys, were dissected and weighed. Bronchoalveolar lavage fluid (BALF) was collected from the right lobe of the lung using phosphate buffered saline (PBS) and the left lobe was weighed and fixed in 10% neutral buffered formalin.

### Hematological and blood biochemical analyses

From the anaesthetized rats, 0.5 mL of blood collected from the abdominal artery was added to a blood collection tube containing an anticoagulant (EDTA-2 K) and analyzed using a hematology analyzer (ADVIA 2120i, Siemens, Munich, Germany). Plasma was separated from the collected blood by placing 1 ml in a tube containing 3.2% sodium citrate and centrifuging at 450 g for 10 min. A coagulation analyzer (ACL ELITE, Werfen Company, Bedford, MA, USA) was used in the blood coagulation test. The following parameters were analyzed: the total count of white blood cell (WBC) count, absolute and relative counts of differential WBC, red blood cell (RBC) count, absolute and relative counts of reticulocytes, hemoglobin (Hb) level, hematocrit (HCT) level, mean corpuscular volume (MCV), mean corpuscular hemoglobin (MCH) level, mean corpuscular hemoglobin concentration (MCHC), platelet (PLT) count, prothrombin time (PT), and activated partial thromboplastin time (APTT).

For the blood biochemistry test, the blood samples except those used for the hematological analyses were added to a tube without anticoagulant, and the samples were placed at 20 °C for 90 min or longer. The samples were then centrifuged at 450 *g* for 10 min to separate the serum. The following parameters were measured using a blood biochemical analyzer (TBA-120FR; Toshiba Co., Tochigi, Japan): the level of glucose, blood urea nitrogen, total protein, albumin, creatinine, total cholesterol, triglyceride (TG), total bilirubin, potassium (K), calcium (Ca), chloride (Cl), inorganic P, and Na; activity of γ-glutamyl transferase, lactate dehydrogenase, creatinine phosphokinase (CK), aspartate aminotransferase, alanine aminotransferase (ALT), and alkaline phosphatase (ALP); and the ratio of albumin-to-globulin.

### Histopathological analysis

The extracted organs were fixed in 10% neutral-buffered formalin and embedded in paraffin. The embedded tissue blocks were cut into 3-μm thick sections and stained with hematoxylin and eosin. The stained sections were examined using a light microscope (Axio Scope A1, 07745 Jena, Germany), and the nomenclature of histopathological assessments is described according to reference 11.

### Bronchoalveolar lavage fluid analysis and cytokines

The bronchoalveolar lavage fluid was analyzed only in male rats 2 weeks after exposure and 4 weeks of recovery. To obtain BALF, the upper end of the trachea was cut and a polypropylene tube attached to a syringe was inserted, and then the trachea was washed three times with 4 mL of PBS. The collected BALF was centrifuged at 450 *g* for 10 min and the supernatant was stored at − 80 °C. The cell pellet was re-suspended in fresh PBS and the total immune cell count was determined using a hematology analyzer (ADVIA 2120i). The re-suspended cell pellet was centrifuged at 270 *g* for 10 min using a cytospin centrifuge (Cellspin; Hanil, Gimpo, Korea) and stained using Diff-Quick staining solution. The differential cell counts were determined using a light microscope at 100× magnification.

### Cytokine analysis

The supernatant separated from the BALF was thawed at around 20 °C just before the cytokine analysis. A commercially available cytokine multi-magnetic bead array kit (R & amp; D Systems, Minneapolis, MN 55413) was used to analyze the concentration of interleukin (IL)-1β, IL-6, IL-4, tumor necrosis factor α (TNF-α), and macrophage inflammatory protein 2-alpha (MIP-2) in the BALF. The Magnetic Bead Single Plex Kit (MILLIPLEX MAP; Merck Millipore, Darmstadt, Germany) was used to measure the concentration of transforming growth factor β (TGF-β). ROS/RNS was analyzed using an OxiSelect™ In Vitro ROS/RNS Assay Kit (Catalog No. STA-347; Cell Biolab, Inc., USA) and Varioskan Flash Reader (Thermo Fisher Scientific, Finland). The assays were performed per the manufacturers’ instructions. The median fluorescence intensity (MFI) of the samples was measured using a Luminex 100 instrument (Luminex, Austin, TX, USA) and a standard curve was obtained using MasterPlex software (MasterPlex QT 2010; Miraibio, Hitachi, CA, USA). The cytokine concentration was calculated using the standard curve.

### Statistical analysis

The data are presented as mean and standard deviation. The data were statistically analyzed using PRISTIMA version 7.1.0 (Xybion Medical Systems Corporation, Morris Plains, NJ, USA). Levene test was performed to determine the homogeneity of variance. When the variance was homogeneous, the one-way analysis of variance was performed, and statistical differences between the control and test groups were analyzed using Dunnett’s test. When the variance was heterogeneous, Kruskal-Wallis test was performed, and statistical differences between the control and test groups were analyzed using Dunn’s rank sum test(*p < 0.05, **p < 0.01). In addition, the benchmark dose (BMD) was calculated using PROAST software version 65.2 provided (www.proast.nl) by the Dutch National Institute for Public Health and the Environment (RIVM).

## Supplementary information

**Additional file 1: Table S1.** Changes in Hematological Parameters–Main group (Rats exposed to BAC for 2 weeks). **Table S2.** Changes in Hematological Parameters–Recovery group (Rats after 2 and 4 week recovery period). **Table S3.** Changes in Serum Chemical Parameters–Main group (Rats exposed to BAC for 2 weeks). **Table S4.** Changes in Serum Chemical Parameters–Recovery group (Rats after 2 and 4 week recovery period). **Table S5.** Histopathological assessment of the liver and spleen tissues.

## Data Availability

The datasets used and/or analyzed during the current study are available from the corresponding author on reasonable request.

## Abbreviations

ALP: Alkaline phosphatase
ALT: Alanine aminotransferase
APTT: Activated partial thromboplastin time
APTT: Activated partial thromboplastin time
BAC: Benzalkonium chloride
BALF: Bronchoalveolar lavage fluid
BAS%: Relative count of basophil
BASA: Absolute count of basophil
CK: Creatinine phosphokinase
EOS%: Relative count of eosinophil
EOSA: Absolute count of eosinophil
F344: Fischer 344 rat
GSD: Geometric standard deviation
Hb: Hemoglobin
HCT: Hematocrit
IL: Interleukin
LYM%: Relative count of lymphocyte
LYMA: Absolute count of lymphocyte
MCH: Mean corpuscular hemoglobin
MCHC: Mean corpuscular hemoglobin concentration
MCV: Mean corpuscular volume
MFI: Median fluorescence intensity
MIP-2: Macrophage inflammatory protein 2-alpha
MMAD: Mass median aerodynamic diameter
MON%: Relative count of monocyte
MONA: Absolute count of monocyte
NEU%: Relative count of neutrophil
NEUA: Absolute count of neutrophil
PBS: Phosphate buffered saline
PLT: Platelet
PT: Prothrombin time
RBC: Red blood cell
RET%: Relative count of reticulocyte
RETA: Absolute count of reticulocyte
RNS: Reactive nitrogen species
ROS: Reactive oxygen species
T1: 0.8 mg/m^3^ BAC exposure group
T2: 4 mg/m^3^ BAC exposure group
T3: 20 mg/m^3^ BAC exposure group
TG: Triglyceride
TGF-β: Transforming growth factor β.
TNF-α: Tumor necrosis factor α
WBC: White blood cell

## References

[1] Food and Drug Administration. 42912 Federal Register/Vol. 81, No. 126/Thrsday, June 30, 2016/Proposed Rules.

[2] European Commission. EU Reference Laboratory for Pesticides Requiring Single Residue Methods: Analysis of Quaternary Ammonium Compounds (QACs) in Fruits and Vegetables using QuEChERS and LC-MS/MS. Version 5, 2016.

[3] Lavorgna M, Russo C, D'Abrosca B, Parrella A, Isidori M (2016). Toxicity and genotoxicity of the quaternary ammonium compound benzalkonium chloride (BAC) using Daphnia magna and Ceriodaphnia dubia as model systems. Environ Pollut.

[4] Prince SJ, McLaury HJ, Allen LV, McLaury P (1999). Analysis of Benzalkonium chloride and its homologs: HPLC versus HPCE. J Pharm Biomed Anal.

[5] Bradosol*.* Archived from the original on 2014-10-12*.* Retrieved 2013-05-20*.*

[6] Ash M, Ash I. Handbook of Preservatives: Synapse Info Resources; 2004. p. 286. ISBN 978-1-890-59566-1.

[7] Nelson L, Goldfrank L. Goldfrank's Toxicologic Emergencies. 9th ed: McGraw-Hill Medical; 2011. p. 803. ISBN 978-1-890-59566-1.

[8] Baudouin C*,* Creuzot-Garcher C, Hoang-Xuan, T. Inflammatory diseases of the conjunctiva (1, illustrated ed.). Thieme*;* 2001. p. 141. ISBN 978-3-131-25871-7.

[9] Malo J*,* Chan-Yeung M, Bernstein DI. Asthma in the Workplace (4, illustrated, revised ed.). CRC Press; 2013. p. 198.

[10] Chungsik Y, Donguk P, Seonggyun K, Sangmin L, Jeonghyeon K, Miyeon S, Jongbo K, Gyujin H, Gyeongmin L, Seonguk Y. Evaluation of health and hazard and survey of workplace use and exposure of biocide aerosol. In: Korea Occupational Safety and Health Institute: Research Report; 2012. p. 13–5.

[11] Roger R, Amy B, Jack H, Ron H, Birgit K, David L, Thomas M, Kasuke N, Michale P, Susanne R, Martin R, Pierre T, Thomas W (2009). Proliferative and nonproliferative lesions of the rat and mouse respiratory tract. Toxicol Pathol.

[12] O’Neil M, Smith A, Heckelman P (2006). The Merck Index: An encyclopedia of chemicals. drugs and biologicals 13th ed.

[13] Gardner WP, Girard JE (2000). Analysis of common household cleaner-disinfectants by capillary electrophoresis. J Chem Educ.

[14] Gilbert P, Moore LE (2005). Cationic antiseptics: diversity of action under a common epithet. J Appl Microbiol.

[15] Ioannou CJ, Hanlon GW, Denyer SP (2007). Action of disinfectant quaternary ammonium compounds against Staphylococcus aureus. Antimicrob Agents Chemother.

[16] Krogsrud NE, Larsen AI (1997). Airborne irritant contact dermatitis from benzalkonium chloride. Contact Dermatitis.

[17] Oiso N, Fukai K, Ishii M (2005). Irritant contact dermatitis from benzalkonium chloride in shampoo. Contact Dermatitis.

[18] Mauleón C, Mauleón P, Chavarría E, De La Cueva P, Suárez R, Pablo L (2006). Airborne contact dermatitis from n-alkyl dimethylbenzylammonium chloride and n-alkyldimethyl- ethyl-benzylammonium chloride in a detergent. Contact Dermatitis.

[19] URL:http://scorecard.goodguide.com. Accessed Feb 2015.

[20] Deutschle T, Porkert U, Reiter R, Keck T, Riechelmann H (2006). Riechelmann, in vitro genotoxicity and cytotoxicity of benzalkonium chloride. Toxicol in Vitro.

[21] Świercz R, Hałatek T, Wąsowicz W, Kur B, Grzelińska Z, Majcherek W (2008). Pulmonary irritation after inhalation exposure to benzalkonium chloride in rats. Int J Occup Med Environ Health.

[22] Świercz R, Hałatek T, Stetkiewicz J, Wąsowicz W, Kur B, Grzelińska Z, Majcherek W (2013). Toxic effect in the lungs of rats after inhalation exposure to benzalkonium chloride. Int J Occup Med Environ Health.

[23] Xue Y, Hieda Y, Kimura K, Takayama K, Fujihara J, Tsujino Y (2004). Kinetic characteristics and toxic effects of benzalkonium chloride following intravascular and oral administration in rats. J Chromatogr B Anal Technol Biomed Life Sci.

[24] Xue Y, Hieda Y, Saito Y, Nomura T, Fujihara J, Takayama K, Kimura K, Takeshita H (2004). Distribution and disposition of benzalkonium chloride following various routes of administration in rats. Toxicol Lett.

[25] OECD (2018). OECD Guidelines on the Testing of Chemicals: Test Guideline 412 Subacute Inhalation Toxicity: 28-Day Study.

[26] Jeffrey JW (2007). Parasympathetic control of airway submucosal glands: central reflexes and the airway intrinsic nervous system. Auton Neurosci.

[27] Evans GO (2009). Erythrocyte, Anemias, and polycythemias. Animal hematotoxicology: A practical guide for toxicologist and biomedical researchers.

[28] Haschek WM, Rousseaux CG, Walling MA (2009). Clinical pathology. Fundamentals of toxicologic pathology.

[29] Asanuma F, Miyata H, Iwaki Y, Kimura M. Feature on erythropoiesis in dietary restricted rats. J Vet Med Sci. 2011. p. 73–89. 10.1292/jvms.10-0175 20823660

[30] Faqi AS (2013). Clinical pathology. A comprehensive guide to toxicology in preclinical drug development.

[31] Xing Z, Gauldie J, Cox G, Baumann H, Jordana M, Lei XF, Achong MK (1998). IL-6 is an antiinflammatory cytokine required for controlling local or systemic acute inflammatory responses. J Clin Invest.

[32] Lee EG, Mickle-Kawar BM, Gallucci RM (2013). IL-6 deficiency exacerbates skin inflammation in a murine model of irritant dermatitis. J Immunol.

[33] Thoolen B, Maronpot RR, Harada T, Nyska A, Rousseaux C, Nolte T, Malarkey DE, Kaufmann W, Küttler K, Deschl U, Nakae D (2010). Proliferative and nonproliferative lesions of the rat and mouse hepatobiliary system. Toxicol Pathol.

[34] Suttie AW (2006). Histopathology of the spleen. Toxicol Pathol.

[35] Lewis RW, Billington R, Debryune E, Gamer A, Lang B, Carpanini F (2002). Recognition of adverse and nonadverse effects in toxicity studies. Toxicol Pathol.

[36] Pearse G (2006). Histopathology of the thymus. Toxicol Pathol.

[37] Harkema JR, Carey SA, Wagner JG (2006). The nose revisited: a brief review of the comparative structure, function, and toxicologic pathology of the nasal epithelium. Toxicol Pathol.

[38] Fokkens WJ, Scheeren RA (2000). Upper airway defence mechanisms. Paediatr Respir Rev.

[39] Nathan RA, Eccles R, Howarth PH, Steinsvåg SK, Togias A (2005). Objective monitoring of nasal patency and nasal physiology in rhinitis. J Allergy Clin Immunol.

[40] Jiao N, Meng L, Zhang I (2014). The effect of topical corticosteroids, topical antihistamines, and preservatives on human ciliary beat frequency. J Otorhinolaryngol Relat Spec.

[41] Mallants R, Jorissen M, Augustijns P (2007). Effect of preservatives on ciliary beat frequency in human nasal epithelial cell culture: single versus multiple exposure. Int J Pharm.

[42] Rizzo JA, Medeiros D, Silva AR, Sarinho E (2006). Benzalkonium chloride and nasal mucociliary clearance: a randomized, placebo-controlled, crossover, double-blind trial. Am J Rhinol.

[43] Lewis RJ (2014). Sax’s Dangerous Properties of Industrial Materials.

[44] “TOXNET Benzalkonium Chloride Compounds”.

[45] “Haz-Map Benzalkonium Chloride”.

[46] “NIOSH ICSC Benzalkonium Chloride”. Archived from the original on 2017-11-16. Retrieved 2017-09-08.

[47] Kuboyama Y, Suzuki K, Hara T (1997). Nasal lesions induced by intranasal administration of benzaikonium chloride in rats. J Toxicol Sci.

[48] Graf P, Hallén H, Juto JE (1995). Benzalkonium chloride in a decongestant nasal spray aggravates rhinitis medicamentosa in healthy volunteers. Clin Exp Allergy.

[49] Graf P, Enerdal J, Hallén H (1999). Ten days' use of oxymetazoline nasal spray with or without benzalkonium chloride in patients with vasomotor rhinitis. Arch Otolaryngol Head Neck Surg.

[50] Graf P, Hallén H (1996). Effect on the nasal mucosa of long-term treatment with oxymetazoline, benzalkonium chloride, and placebo nasal sprays. Laryngoscope.

[51] Kwon DY, Kwon JT, Lim YM, Shim IS, Kim EJ, Lee DH, Yoon BI, Kim PJ, Kim HM (2019). Inhalation toxicity of benzalkonium chloride and triethylene glycol mixture in rats. Toxicol Appl Pharmacol.

[52] ECHA (2012). Guidance on information requirements and chemical safety assessment Chapter R.8: Characterisation of Dose [concentration]-response for human health, Version 2.

[53] Lee JH, Kim YH, Kwon JH (2012). Fatal misuse of humidifier disinfectants in Korea: importance of screening risk assessment and implications for management of chemicals in consumer products. Environ Sci Technol.

