# Supplementary material for: Assessment of respiratory and systemic toxicity of Benzalkonium chloride following a 14-day inhalation study in rats
Source: Part Fibre Toxicol. 2020 Jan 28;17:5. doi: 10.1186/s12989-020-0339-8 (PMC6986023; doi:10.1186/s12989-020-0339-8)
Supplement: Supplementary file 1 — Additional file 1: Table S1. Changes in Hematological Parameters–Main group (Rats exposed to BAC for 2 weeks). Table S2. Changes in Hematological Parameters–Recovery group (Rats after 2 and 4 week recovery period). Table S3. Changes in Serum Chemical Parameters–Main group (Rats exposed to BAC for 2 weeks). Table S4. Changes in Serum Chemical Parameters–Recovery group (Rats after 2 and 4 week recovery period). Table S5. Histopathological assessment of the liver and spleen tissues. [file 12989_2020_339_MOESM1_ESM.docx]

**Additional file**

**Table S1.** Changes in Hematological Parameters – Main group

| Parameters | Male | | | | Female | | | |
| --- | --- | --- | --- | --- | --- | --- | --- | --- |
|  | Control | 0.8 mg/m^3^ | 4 mg/m^3^ | 20 mg/m^3^ | Control | 0.8 mg/m^3^ | 4 mg/m^3^ | 20 mg/m^3^ |
| RBC(x10^6^/µL) | 8.40  ±0.29 | 8.68  ±0.16 | 8.97  ±0.13** | 9.56  ±0.19** | 9.04  ±0.21 | 8.82  ±0.10 | 9.21  ±0.15 | 9.54  ±0.37 |
| HCT(%) | 43.52  ±1.27 | 44.56  ±1.17 | 45.42  ±0.69* | 48.12  ±1.16** | 45.28  ±0.59 | 44.48  ±0.61 | 46.12  ±0.76 | 47.58  ±2.35 |
| HGB(g/dL) | 14.50  ±0.26 | 14.96  ±0.21* | 15.42  ±0.30** | 16.44  ±0.34** | 15.60  ±0.24 | 15.32  ±0.42 | 15.86  ±0.23 | 16.40  ±0.67 |
| MCV(fL) | 51.84  ±1.06 | 51.30  ±0.57 | 50.62  ±0.59 | 50.32  ±0.37* | 50.14  ±0.67 | 50.40  ±0.46 | 50.06  ±0.39 | 59.88  ±0.51 |
| MCH(g/dL) | 17.28  ±0.37 | 17.24  ±0.15 | 17.20  ±0.37 | 17.22  ±0.16 | 17.28  ±0.28 | 17.38  ±0.35 | 17.20  ±0.10 | 17.20  ±0.07 |
| MCHC(g/dL) | 33.28  ±0.52 | 33.62  ±0.50 | 33.96  ±0.49 | 34.16  ±0.37* | 34.46  ±0.25 | 34.44  ±0.72 | 34.36  ±0.17 | 34.46  ±0.39 |
| PLT(10³/µL) | 928  ±39 | 892  ±23 | 823  ±43** | 510  ±39** | 841  ±50 | 806  ±47 | 844  ±79 | 843  ±73 |
| WBC(x10³/µL) | 4.17  ±0.73 | 4.56  ±0.46 | 3.47  ±0.72 | 3.07  ±0.84 | 4.42  ±0.59 | 4.09  ±0.54 | 3.55  ±0.60 | 2.50  ±0.39** |
| NEUA(10³/µL) | 0.78  ±0.25 | 0.78  ±0.21 | 0.82  ±0.22 | 0.70  ±0.21 | 0.81  ±0.16 | 0.67  ±0.11 | 0.67  ±0.24 | 0.56  ±0.21 |
| NEU%(%) | 18.50  ±3.68 | 17.00  ±4.52 | 24.04  ±6.28 | 22.86  ±5.70 | 18.48  ±2.83 | 16.62  ±3.90 | 19.50  ±7.59 | 22.22  ±5.04 |
| LYMA(10³/µL) | 3.22  ±0.51 | 3.58  ±0.41 | 2.45  ±0.58 | 2.23  ±0.67* | 3.35  ±0.51 | 3.22  ±0.58 | 2.68  ±0.64 | 1.78  ±0.19** |
| LYM%(%) | 77.48  ±3.94 | 78.38  ±4.49 | 70.62  ±6.50 | 72.36  ±5.60 | 75.82  ±3.11 | 78.14  ±5.33 | 75.00  ±8.21 | 71.64  ±5.39 |
| MONA(10³/µL) | 0.08  ±0.02 | 0.10  ±0.01 | 0.07  ±0.03 | 0.07  ±0.04 | 0.13  ±0.03 | 0.09  ±0.01 | 0.09  ±0.04 | 0.07  ±0.02** |
| MON%(%) | 1.94  ±2.40 | 2.14  ±0.23 | 2.14  ±0.45 | 2.20  ±0.70 | 3.06  ±0.28 | 2.24  ±0.36 | 2.44  ±1.01 | 2.66  ±0.53 |
| EOSA(10³/µL) | 0.05  ±0.01 | 0.07  ±0.02 | 0.08  ±0.02* | 0.05  ±0.02 | 0.06  ±0.01 | 0.07  ±0.03 | 0.07  ±0.02 | 0.06  ±0.01 |
| EOS%(%) | 1.20  ±0.27 | 1.46  ±0.26 | 2.34  ±0.23* | 1.80  ±0.90 | 1.40  ±0.25 | 1.78  ±0.90 | 2.02  ±0.31 | 2.38  ±0.30 |
| BASA(10³/µL) | 0.01  ±0.00 | 0.01  ±0.00 | 0.01  ±0.01 | 0.00  ±0.01 | 0.01  ±0.01 | 0.01  ±0.00 | 0.01  ±0.00 | 0.00  ±0.01 |
| BAS%(%) | 0.32  ±0.05 | 0.26  ±0.09 | 0.18  ±0.08* | 0.18  ±0.08* | 0.30  ±0.14 | 0.32  ±0.05 | 0.22  ±0.08 | 0.26  ±0.18 |
| RETA(10^9^/L) | 260.70  ±41.33 | 241.48  ±22.83 | 190.72  ±17.52 | 30.48  ±2.94** | 186.50  ±40.82 | 162.16  ±28.66 | 139.66  ±14.54 | 137.66  ±15.18 |
| RET%(%) | 3.12  ±0.58 | 2.79  ±0.29 | 2.12  ±0.20 | 0.32  ±0.03** | 2.07  ±0.50 | 1.84  ±0.33 | 1.52  ±0.18 | 1.44  ±0.12* |
| APTT(sec) | 16.26  ±0.67 | 17.10  ±0.55 | 18.12  ±0.66 | 21.08  ±1.89** | 17.44  ±1.46 | 16.88  ±0.51 | 17.78  ±0.70 | 19.32  ±0.92* |
| PT(sec) | 11.00  ±0.38 | 10.98  ±0.61 | 12.04  ±0.42* | 12.78  ±0.68** | 11.18  ±0.37 | 11.58  ±0.30 | 12.64  ±0.54** | 13.70  ±0.41** |

RBC, red blood cell count; HCT, hematocrit; HGB, hemoglobin; MCV, mean corpuscular volume; MCH, mean corpuscular hemoglobin; MCHC, mean corpuscular hemoglobin concentration; PLT, platelet; WBC, white blood cell count; NEUA, absolute count of neutrophil; NEU%, relative count of neutrophil; LYMA, absolute count of lymphocyte; LYM%, relative count of lymphocyte; MONA, absolute count of monocyte; MON%, relative count of monocyte EOSA, absolute count of eosinophil; EOS%, relative count of eosinophil; BASA, absolute count of basophil; BAS%, relative count of basophil; RET, absolute count of reticulocyte; RET%, relative count of reticulocyte; APTT, Activated partial thromboplastin time; PT, Prothrombin time. The values are expressed as mean ± SD (n = 5 per group). * represents statistical significance as compared to the control group, p <0.05. ** represents statistical significance as compared to the control group, p <0.01.

**Table S2.** Changes in Hematological Parameters – Recovery group

| Parameters | 2 Weeks | | | | 4 Weeks | | | |
| --- | --- | --- | --- | --- | --- | --- | --- | --- |
|  | Control | 0.8 mg/m^3^ | 4 mg/m^3^ | 20 mg/m^3^ | Control | 0.8 mg/m^3^ | 4 mg/m^3^ | 20 mg/m^3^ |
| RBC(x10^6^/µL) | 9.02  ±0.31 | 8.98  ±0.12 | 8.96  ±0.27 | 8.95  ±0.16 | 8.89  ±0.10 | 9.00  ±0.19 | 8.89  ±0.16 | 8.77  ±0.31 |
| HCT(%) | 43.68  ±1.37 | 43.40  ±0.68 | 43.66  ±0.84 | 44.22  ±0.93 | 42.16  ±0.26 | 42.62  ±0.66 | 42.52  ±0.44 | 42.78  ±1.71 |
| HGB(g/dL) | 14.84  ±0.47 | 14.72  ±0.30 | 14.72  ±0.34 | 14.90  ±0.29 | 14.28  ±0.24 | 14.46  ±0.22 | 14.38  ±0.15 | 14.38  ±0.50 |
| MCV(fL) | 48.42  ±0.36 | 48.32  ±0.36 | 48.80  ±0.68 | 49.38  ±0.21** | 47.40  ±0.39 | 47.32  ±0.40 | 47.84  ±0.54 | 48.74  ±0.55** |
| MCH(g/dL) | 16.4  ±0.1 | 16.4  ±0.2 | 16.4  ±0.2 | 16.7  ±0.1 | 16.0  ±0.1 | 16.1  ±0.1 | 16.1  ±0.3 | 16.4  ±0.1** |
| MCHC(g/dL) | 33.91  ±0.07 | 33.94  ±0.25 | 33.70  ±0.30 | 33.74  ±0.30 | 33.92  ±0.43 | 33.96  ±0.13 | 33.78  ±0.31 | 33.60  ±0.27 |
| PLT(10³/µL) | 788.0  ±37.29 | 816.4  ±11.78 | 871.4  ±32.55** | 872.2  ±21.35** | 787  ±20 | 773  ±43 | 806  ±36 | 789  ±49 |
| WBC(x10³/µL) | 4.65  ±1.10 | 4.65  ±0.50 | 4.48  ±0.92 | 4.64  ±0.71 | 4.70  ±0.46 | 5.38  ±0.69 | 4.45  ±0.54 | 3.74  ±1.15 |
| NEUA(10³/µL) | 1.19  ±0.30 | 1.07  ±0.13 | 1.14  ±0.14 | 1.12  ±0.27 | 1.08  ±0.19 | 1.25  ±0.45 | 1.34  ±0.54 | 1.01  ±0.30 |
| NEU%(%) | 25.90  ±4.84 | 22.92  ±0.85 | 26.04  ±4.42 | 23.94  ±3.42 | 23.16  ±4.30 | 22.84  ±5.10 | 30.38  ±12.10 | 27.72  ±5.46 |
| LYMA(10³/µL) | 3.23  ±0.87 | 3.37  ±0.37 | 3.13  ±0.84 | 3.29  ±0.42 | 3.40  ±0.44 | 3.89  ±0.27 | 2.89  ±0.72 | 2.58  ±0.87 |
| LYM%(%) | 69.38  ±5.45 | 72.52  ±1.23 | 69.22  ±4.63 | 71.36  ±3.86 | 72.32  ±4.70 | 72.92  ±5.37 | 64.78  ±12.30 | 68.02  ±6.01 |
| MONA(10³/µL) | 0.12  ±0.05 | 0.10  ±0.37 | 0.11  ±0.03 | 0.13  ±0.05 | 0.11  ±0.03 | 0.12  ±0.04 | 0.12  ±0.02 | 0.08  ±0.03 |
| MON%(%) | 2.48  ±0.64 | 2.22  ±0.55 | 2.46  ±0.48 | 2.62  ±0.74 | 2.40  ±0.64 | 2.12  ±0.40 | 2.70  ±0.46 | 2.12  ±0.58 |
| EOSA(10³/µL) | 0.07  ±0.01 | 0.07  ±0.01 | 0.06  ±0.01 | 0.07  ±0.02 | 0.07  ±0.03 | 0.08  ±0.02 | 0.06  ±0.02 | 0.06  ±0.01 |
| EOS%(%) | 1.44  ±0.21 | 1.52  ±0.26 | 1.28  ±0.30 | 1.48  ±0.16 | 1.48  ±0.48 | 1.48  ±0.31 | 1.42  ±0.43 | 1.68  ±0.31 |
| BASA(10³/µL) | 0.01  ±0.01 | 0.01  ±0.01 | 0.01  ±0.01 | 0.01  ±0.00 | 0.01  ±0.01 | 0.01  ±0.01 | 0.01  ±0.01 | 0.01  ±0.00 |
| BAS%(%) | 0.16  ±0.06 | 0.24  ±0.11 | 0.16  ±0.09 | 0.14  ±0.06 | 0.20  ±0.10 | 0.14  ±0.13 | 0.16  ±0.06 | 0.18  ±0.08 |
| RETA(10^9^/L) | 250.48  ±34.86 | 256.74  ±5.45 | 328.56  ±47.76** | 319.96  ±30.79* | 270.34  ±16.42 | 239.50  ±18.57 | 280.40  ±25.11 | 292.90  ±20.89 |
| RET%(%) | 2.78  ±0.40 | 2.86  ±0.05 | 3.67  ±0.58** | 3.58  ±0.33* | 3.04  ±0.18 | 2.66  ±0.25 | 3.15  ±0.32 | 3.34  ±0.18 |
| APTT(sec) | 19.48  ±2.32 | 18.92  ±0.66 | 19.12  ±0.35 | 21.04  ±3.60 | 16.54  ±0.68 | 17.22  ±0.42 | 16.70  ±0.44 | 16.90  ±0.57 |
| PT(sec) | 10.28  ±0.16 | 10.72  ±0.52 | 9.98  ±0.11 | 10.24  ±0.24 | 10.46  ±0.35 | 10.68  ±0.26 | 10.38  ±0.42 | 11.42  ±0.46** |

RBC, red blood cell count; HCT, hematocrit; HGB, hemoglobin; MCV, mean corpuscular volume; MCH, mean corpuscular hemoglobin; MCHC, mean corpuscular hemoglobin concentration; PLT, platelet; WBC, white blood cell count; NEUA, absolute count of neutrophil; NEU%, relative count of neutrophil; LYMA, absolute count of lymphocyte; LYM%, relative count of lymphocyte; MONA, absolute count of monocyte; MON%, relative count of monocyte EOSA, absolute count of eosinophil; EOS%, relative count of eosinophil; BASA, absolute count of basophil; BAS%, relative count of basophil; RET, absolute count of reticulocyte; RET%, relative count of reticulocyte; APTT, Activated partial thromboplastin time; PT, Prothrombin time. The values are expressed as mean ± SD (n = 5 per group). * represents statistical significance as compared to the control group, p <0.05. ** represents statistical significance as compared to the control group, p <0.01.

**Table S3.** Changes in Serum Chemical Parameters – Main group

| Parameters | Male | | | | Female | | | |
| --- | --- | --- | --- | --- | --- | --- | --- | --- |
|  | Control | 0.8 mg/m^3^ | 4 mg/m^3^ | 20 mg/m^3^ | Control | 0.8 mg/m^3^ | 4 mg/m^3^ | 20 mg/m^3^ |
| Ca(mg/dL) | 9.04  ±0.21 | 9.00  ±0.14 | 9.08  ±0.27 | 8.98  ±0.34 | 9.06  ±0.17 | 9.14  ±0.17 | 8.90  ±0.29 | 9.28  ±0.34 |
| IP(mg/dL) | 7.80  ±0.61 | 7.74  ±0.57 | 7.50  ±0.30 | 7.66  ±0.35 | 7.14  ±0.57 | 7.02  ±0.59 | 7.30  ±0.24 | 7.30  ±0.57 |
| ALP(IU/L) | 914.28  ±71.49 | 896.70  ±51.47 | 788.74  ±45.07* | 675.46  ±74.70** | 599.54  ±44.44 | 592.44  ±52.97 | 647.14  ±21.79 | 639.04  ±74.65 |
| ALT(IU/L) | 36.06  ±3.04 | 37.28  ±3.52 | 35.24  ±2.64 | 47.22  ±11.48* | 30.14  ±2.59 | 28.02  ±3.39 | 32.34  ±3.24 | 36.52  ±5.64 |
| AST(IU/L) | 108.62  ±10.87 | 111.42  ±10.47 | 105.28  ±11.81 | 102.74  ±15.07 | 79.62  ±6.63 | 85.82  ±13.73 | 87.77  ±7.52 | 81.00  ±3.03 |
| TG(mg/dL) | 53.98  ±20.09 | 49.26  ±9.06 | 27.02  ±6.12 | 17.92  ±1.44** | 16.76  ±3.52 | 16.26  ±5.62 | 12.88  ±2.39 | 16.56  ±2.96 |
| TCHO(mg/dL) | 46.12  ±4.76 | 47.68  ±2.69 | 50.20  ±4.72 | 55.60  ±9.15 | 61.30  ±4.51 | 62.36  ±2.68 | 59.00  ±3.62 | 60.64  ±6.22 |
| TBIL(mg/dL) | 0.17  ±0.02 | 0.18  ±0.03 | 0.19  ±0.01 | 0.13  ±0.03 | 0.13  ±0.01 | 0.12  ±0.01 | 0.13  ±0.01 | 0.13  ±0.01 |
| GLU(mg/dL) | 122.78  ±28.49 | 123.68  ±20.62 | 117.06  ±13.88 | 90.28  ±15.66 | 88.70  ±3.99 | 92.94  ±11.01 | 91.50  ±12.50 | 83.22  ±9.96 |
| CREA(mg/dL) | 0.35  ±0.03 | 0.35  ±0.03 | 0.34  ±0.16 | 0.33  ±0.02 | 0.34  ±0.02 | 0.35  ±0.03 | 0.36  ±0.03 | 0.35  ±0.02 |
| BUN(mg/dL) | 17.72  ±2.39 | 16.70  ±1.19 | 15.12  ±1.26 | 19.58  ±2.57 | 16.16  ±1.00 | 16.48  ±1.88 | 15.14  ±2.52 | 16.46  ±1.43 |
| ALB(g/dL) | 3.64  ±0.09 | 3.74  ±0.09 | 3.74  ±0.09 | 3.64  ±0.06 | 3.72  ±0.15 | 3.68  ±0.13 | 3.66  ±0.06 | 3.86  ±0.11 |
| TP(g/dL) | 5.16  ±0.17 | 5.28  ±0.15 | 5.34  ±0.18 | 5.06  ±0.11 | 5.34  ±0.30 | 5.22  ±0.16 | 2.24  ±0.11 | 5.58  ±0.24 |
| Cl(mmol/L) | 94.62  ±0.71 | 96.32  ±3.05 | 95.56  ±0.20 | 91.06  ±2.88 | 97.62  ±1.40 | 97.78  ±0.61 | 99.04  ±0.63 | 97.32  ±1.15 |
| K(mmol/L) | 4.52  ±0.22 | 4.62  ±0.13 | 4.80  ±0.19 | 4.64  ±0.37 | 4.08  ±0.16 | 4.06  ±0.11 | 4.32  ±0.15* | 4.26  ±0.13 |
| Na(mmol/L) | 134.70  ±0.49 | 135.72  ±2.96 | 134.98  ±0.67 | 136.34  ±1.176 | 134.58  ±0.52 | 135.22  ±0.61 | 136.80  ±0.83** | 138.10  ±0.77** |
| CK(U/L) | 618.66  ±142.76 | 583.08  ±163.15 | 545.66  ±130.72 | 244.94  ±41.77** | 242.06  ±35.26 | 297.80  ±148.68 | 257.30  ±106.39 | 212.24  ±63.77 |
| LDH (IU/L) | 2580.78  ±530.41 | 2600.72  ±521.65 | 2530.12  ±436.79 | 1859.44  ±403.91 | 902.32  ±124.11 | 1285.04  ±698.89 | 1259.58  ±572.66 | 1026.60  ±326.83 |
| γ-GTP(IU/L) | 0.00  ±0.12 | 0.53  ±0.98 | 0.00  ±0.26 | 0.00  ±0.25 | 0.16  ±0.33 | -0.20  ±0.21 | -0.27  ±0.34 | 0.20  ±0.79 |
| A/G ratio | 2.40  ±0.10 | 2.42  ±0.13 | 2.34  ±0.13 | 2.58  ±0.15 | 2.30  ±0.13 | 2.38  ±0.08 | 2.34  ±0.11 | 2.26  ±0.11 |

Ca, Calcium; IP, Inorganic phosphorus; ALP, alkaline phophatase; ALT, alanine aminotransferase; AST, aspartate aminotransferase; TG, Triglyceride; TCHO, total cholesterol; TBIL, total bilirubin; GLU, glucose; CREA, Creatinine; BUN, blood urea nitrogen; ALB, albumin; TP, total protein; Cl, Chloride; K, Potassium; Na, Sodium; CK, Creatine phosphokinase; LDH, Lactate dehydrogenase; γ-GTP, γ-Glutamyl Transferase; A/G ratio, Albumin/Globulin ratio. The values are expressed as mean ± SD (n = 5 per group). * represents statistical significance as compared to the control group, p <0.05. ** represents statistical significance as compared to the control group, p <0.01.

**Table S4**. Changes in Serum Chemical Parameters – Recovery group

| Parameters | 2 Weeks | | | | 4 Weeks | | | |
| --- | --- | --- | --- | --- | --- | --- | --- | --- |
|  | Control | 0.8 mg/m^3^ | 4 mg/m^3^ | 20 mg/m^3^ | Control | 0.8 mg/m^3^ | 4 mg/m^3^ | 20 mg/m^3^ |
| Ca(mg/dL) | 10.00  ±0.03 | 10.08  ±0.16 | 10.20  ±0.52 | 9.98  ±0.25 | 10.00  ±0.14 | 10.12  ±0.21 | 10.06  ±0.17 | 9.94  ±0.45 |
| IP(mg/dL) | 6.58  ±0.54 | 7.04  ±0.46 | 7.06  ±0.59 | 7.68  0.28** | 6.24  ±0.29 | 6.32  ±0.34 | 6.20  ±0.52 | 6.68  ±0.25 |
| ALP(IU/L) | 695.52  ±28.52 | 688.96  ±29.80 | 777.70  ±51.71* | 770.64  ±54.58* | 594.10  ±24.24 | 579.14  ±23.36 | 648.06  ±40.10 | 680.92  ±62.32* |
| ALT(IU/L) | 49.40  ±23.18 | 39.28  ±2.88 | 40.52  ±1.10 | 42.12  ±2.80 | 41.40  ±1.05 | 43.18  ±1.00 | 42.18  ±1.25 | 45.02  ±6.36 |
| AST(IU/L) | 100.58  ±43.05 | 77.20  ±6.56 | 87.40  ±8.19 | 87.78  ±8.06 | 89.16  ±4.59 | 87.18  ±10.28 | 88.84  ±8.87 | 95.50  ±12.79 |
| TG(mg/dL) | 74.30  ±9.57 | 66.82  ±12.77 | 74.64  ±24.61 | 63.28  ±9.17 | 104.52  ±25.24 | 84.08  ±27.14 | 94.68  ±37.22 | 59.40  ±18.83 |
| TCHO(mg/dL) | 63.52  ±5.03 | 61.30  ±3.74 | 59.26  ±3.75 | 66.48  ±3.47 | 69.72  ±7.38 | 67.42  ±5.92 | 61.86  ±6.25 | 61.60  ±5.31 |
| TBIL(mg/dL) | 0.16  ±0.03 | 0.15  ±0.03 | 0.17  ±0.05 | 0.17  ±0.03 | 0.18  ±0.02 | 0.17  ±0.01 | 0.19  ±0.02 | 0.21  ±0.36 |
| GLU(mg/dL) | 173.02  ±22.57 | 177.10  ±17.59 | 164.04  ±11.25 | 148.70  ±12.37* | 157.30  ±12.05 | 160.40  ±11.32 | 162.64  ±14.06 | 170.60  ±53.29 |
| CREA(mg/dL) | 0.47  ±0.03 | 0.44  ±0.03 | 0.44  ±0.04 | 0.41  ±0.03 | 0.50  ±0.01 | 0.47  ±0.02 | 0.50  ±0.03 | 0.48  ±0.07 |
| BUN(mg/dL) | 22.00  ±1.97 | 19.80  ±1.12 | 19.38  ±1.24* | 18.68  ±1.11** | 21.26  ±1.42 | 19.64  ±1.29 | 19.60  ±0.53 | 19.28  ±2.06 |
| ALB(g/dL) | 4.06  ±0.06 | 3.98  ±0.05 | 4.04  ±0.06 | 3.84  ±0.06** | 4.04  ±0.09 | 3.98  ±0.05 | 4.00  ±0.00 | 3.96  ±0.13 |
| TP(g/dL) | 7.28  ±0.11 | 7.08  ±0.21 | 1.32  ±0.28 | 6.88  ±0.18* | 7.46  ±0.09 | 7.36  ±0.23 | 7.50  ±0.10 | 7.38  ±0.25 |
| Cl(mmol/L) | 102.48  ±1.21 | 103.94  ±0.85 | 104.10  ±0.88 | 103.24  ±1.44 | 102.24  ±1.30 | 103.20  ±1.39 | 103.96  ±0.40 | 104.20  ±0.91* |
| K(mmol/L) | 4.56  ±0.68 | 4.36  ±0.53 | 4.34  ±0.57 | 4.92  ±1.06 | 4.40  ±0.46 | 4.52  ±0.80 | 5.04  ±0.42 | 4.56  ±0.80 |
| Na(mmol/L) | 143.74  ±1.44 | 144.24  ±0.81 | 144.92  ±0.41 | 143.92  ±1.43 | 143.40  ±0.68 | 143.50  ±0.62 | 144.34  ±0.44* | 144.64  ±0.49** |
| CK(U/L) | 311.82  ±119.04 | 270.86  ±127.51 | 298.22  ±125.41 | 290.66  ±107.74 | 453.02  ±114.84 | 400.54  ±191.10 | 384.44  ±151.55 | 435.16  ±225.82 |
| LDH (IU/L) | 889.06  ±435.89 | 753.14  ±459.98 | 858.76  ±439.50 | 842.66  ±419.57 | 1385.62  ±356.50 | 1218.96  ±657.15 | 1237.22  ±515.72 | 1384.60  ±836.99 |
| γ-GTP(IU/L) | 0.00  ±0.20 | 0.16  ±0.50 | 0.04  ±0.33 | 0.22  ±0.80 | 0.00  ±0.20 | 0.08  ±0.29 | 0.00  ±0.38 | 0.00  ±0.49 |
| A/G ratio | 1.26  ±0.06 | 1.30  ±0.07 | 1.22  ±0.11 | 1.30  ±0.07 | 1.18  ±0.05 | 1.20  ±0.10 | 1.14  ±0.06 | 1.16  ±0.06 |

Ca, Calcium; IP, Inorganic phosphorus; ALP, alkaline phophatase; ALT, alanine aminotransferase; AST, aspartate aminotransferase; TG, Triglyceride; TCHO, total cholesterol; TBIL, total bilirubin; GLU, glucose; CREA, Creatinine; BUN, blood urea nitrogen; ALB, albumin; TP, total protein; Cl, Chloride; K, Potassium; Na, Sodium; CK, Creatine phosphokinase; LDH, Lactate dehydrogenase; γ-GTP, γ-Glutamyl Transferase; A/G ratio, Albumin/Globulin ratio. The values are expressed as mean ± SD (n = 5 per group). * represents statistical significance as compared to the control group, p <0.05. ** represents statistical significance as compared to the control group, p <0.01.

**Table S5**. Histopathological assessment of the liver and spleen tissues

|  |  | Main group | | | | | | | | | | |
| --- | --- | --- | --- | --- | --- | --- | --- | --- | --- | --- | --- | --- |
| Sex |  | Male | | | |  | | Female | | | | |
| Concentration (mg/m^3^) |  | 0 | 0.8 | 4 | 20 |  | | 0 | | 0.8 | 4 | 20 |
| Liver |  |  |  |  |  |  | |  | |  |  |  |
| Number of animals |  | 5 | 5 | 5 | 5 |  | | 5 | | 5 | 5 | 5 |
| Atrophy  Minimal  Mild  Moderate  Mean ± SD |  | (0)  0  0  0  0 | (0)  0  0  0  0 | (0)  2  0  0  0 | (5)  1  3  1  2.00 ± 0.71 |  | (0)  0  0  0  0 | | (0)  0  0  0  0 | | (0)  0  0  0  0 | (2)  2  0  0  0.40 ± 0.55 |
| Spleen |  |  |  |  |  |  |  | |  | |  |  |
| Number of animals |  | 5 | 5 | 5 | 5 |  | 5 | | 5 | | 5 | 5 |
| Atrophy  Minimal  Mild  Moderate  Mean ± SD |  | (0)  0  0  0  0 | (0)  0  0  0  0 | (0)  0  0  0  0 | (5)  2  2  1  1.80 ± 0.84 |  | (0)  0  0  0  0 | | (0)  0  0  0  0 | | (0)  0  0  0  0 | (5)  3  2  0  1.40 ± 0.55 |
| Thymus |  |  |  |  |  |  |  | |  | |  |  |
| Number of animals |  | 5 | 5 | 5 | 5 |  | 5 | | 5 | | 5 | 5 |
| Atrophy  Minimal  Mild  Moderate  Marked  Mean ± SD |  | (0)  0  0  0  0  0 | (0)  0  0  0  0  0 | (0)  0  0  0  0  0 | (5)  0  0  4  1  3.20 ± 0.45 |  | (0)  0  0  0  0 | | (0)  0  0  0  0 | | (0)  0  0  0  0 | (3)  2  1  0  0.80 ± 0.84 |

0: unremarkable=no presence of histopathologic lesion; 1: minimal=lesions involving<10% of the tissue of each organ; 2: mild=lesions involving<10-30% of the tissue of each organ; 3: moderate=lesions involving<30-50% of the tissue of each organ; 4: marked=lesions involving<50-70% of the tissue of each organ; 5: severe=lesions involving>70% of the tissue of each organ
